# Endothelial epigenetic senescence driven microglial activation mediates cardio-retinal neuroinflammation in heart failure

**DOI:** 10.7150/thno.136220

**Published:** 2026-07-13

**Authors:** Mengdan Wang, Shuo Zhang, Tursunjan Aziz, Shiyao Zhang, Xiao Wu, Gang Li, Yan Wang

**Affiliations:** 1 Xiamen Cardiovascular Hospital of Xiamen University, School of Medicine, Xiamen University, Xiamen, Fujian 361102, China.; 2 State Key Laboratory of Membrane Biology, Tsinghua-Peking Center for Life Sciences, School of Life Sciences, Tsinghua University, Beijing 100084, China.

**Keywords:** heart failure, microglia activation, neuroinflammation, epigenetic senescence, TGFβ2

## Abstract

**Rationale:**

Heart failure (HF) is increasingly recognized as a systemic disorder that extends beyond the heart and affects neurovascular tissues, including the retina. However, the mechanisms by which circulating factors from HF trigger retinal neuroinflammation remain unclear.

**Methods:**

HF was induced in adult mice by transverse aortic constriction (TAC). Retinal structure and function were evaluated using optical coherence tomography (OCT) and electroretinography (ERG). Parabiosis and plasma transfer experiments were performed to assess the role of circulating factors. Endothelial senescence, microglial activation, and inflammatory signaling were analyzed using immunofluorescence, qPCR, and molecular assays. The functional relevance of TGFβ2 and microglia was tested using anti-TGFβ2 antibody administration and microglial depletion with PLX5622 treatment.

**Results:**

TAC mice exhibited pronounced retinal thinning, diminished electroretinography (ERG) amplitudes, and reduced vascular density. Exposure of healthy mice to HF plasma reproduced these abnormalities, indicating that circulating mediators drive retinal injuries. TGFβ2 levels were markedly elevated in the plasma of both patients with HF and TAC mice. Mechanistically, TGFβ2 activated the pSMAD2/EP300 pathway in retinal endothelial cells, promoting H3K9 acetylation, P21 induction, and endothelial cell senescence. Senescent endothelial cells release proinflammatory factors that activate retinal microglia, leading to hypertrophic morphology, enhanced synaptic phagocytosis, and upregulation of cytokines such as IL1β, TNFα, and IL6. Neutralization of TGFβ2 or microglial depletion markedly reduced inflammation, preserved the retinal architecture, and restored visual function.

**Conclusions:**

Elevated TGFβ2 levels in heart failure drive retinal endothelial epigenetic senescence, which secondarily activates microglia and induces neuroinflammation. Endothelial-specific disruption of TGFβ2 signaling is sufficient to protect the retina independently of primary cardiac recovery. Targeting the TGFβ2–endothelial–microglia axis may represent a promising therapeutic strategy for preventing retinal neurovascular degeneration associated with systemic cardiac disease.

## Introduction

Heart failure (HF) is a major healthcare burden equally affecting an estimated 26 million peopleglobally with an exponentially rising trend 1. Even with emerging therapies, the five-year survival of HF patients is still < 50% 2-6. Heart failure is not a heart disease, but rather a disease of varying severity and severity of function/structure of multiple organs and tissues 7. The cellular stress owing to inflammation and accelerated senescence leads to increased dysfunctional protein production, which enter the circulation and impact distant organs 8-10. Although such findings highlight that HF has a systemic effect, the mechanisms of how circulating factors affect the remote organs, especially with respect to protein misfolding, are poorly understood.

Recent studies have demonstrated that HF affects respiratory and cardiovascular systems. It has also been shown to impair the central nervous system with functions 11-13. The retina is part of the central nervous system. Its embryological origin and vascular supply are similar to the brain. The proper functioning of the immune system depends on the careful regulation of its internal environment 14. The retina’s density, vascular structure, and high metabolic demand make it susceptible to insult from any cardiovascular damage. Retinal microvascular changes are considered the first visible signs of systemic cardiovascular diseases 15, 16. Past studies have shown that HF and atherosclerosis or ischemic heart disease harm retinal vasculature 15, 17-21. Chronic inflammatory response and oxidative damage worsen blood-retinal barrier breakdown and further aggravate vascular pathology 15, 17-21. Even with these findings, the mechanisms which HF interacts with the retinal vasculature system and function is unclear.

In this study, we showed that HF severely damages the retinal and vascular networks in mice. It is worth noting that plasma from HF mice can do similar harm to healthy mice. Therefore, these findings suggest that blood-borne circulating factors are important for HF retinal injury. Further investigation revealed a significantly elevated level of TGFβ2 in the blood of heart failure patients and heart failure mice. TGFβ2 is known for its important functions in regulating cell proliferation, differentiation and apoptosis 22, 23. Within the cardiovascular system, the dysregulated expression of TGFβ2 is tightly associated with the main pathophysiological features of HF, including cardiac remodelling, fibrosis and inflammation 23, 24. Results showed that TGFβ2 activated the phosphorylated SMAD2 (pSMAD2)/EP300 pathway and enhanced the acetylation of histone H3 on lysine 9 (H3K9ac) to activate chromatin remodeling and upregulation of P21. The epigenetic senescence of endothelial cells is promoted by this cascade. Afterward, senescent endothelial cells release senescence-associated secretory phenotype (SASP) factors. This activates retinal microglia, increases their phagocytic activity, amplifies neuroinflammation. Consequently, retinal neurons get damaged in the process. Significantly, depletion of microglia interrupts this pathogenic mechanism, preventing damage to retinal structure and function caused by HF.

To probe the therapeutic utility of inhibiting TGFβ2, HF mice were treated with anti-TGFβ2 antibody by intravitreal injection. The findings displayed a decrease in the aging of endothelial cells, as well as improvement in functionality and structure of the retina. Apart from affecting the retina, our research has indicated that HF can lead to endothelial senescence in various organs, including the spleen, lungs, kidneys, and even the heart. The anti-TGFβ2 antibody given intravenously was similarly effective in reducing endothelial cell senescence in these organs. The antibody significantly reduced cell senescence induced by plasma from HF patients, which was verified on primary human retinal microvascular endothelial cells.

In conclusion, these findings offer new insights into the mechanisms driving HF-induced damage to the retina and other organs. They further provide a theoretical basis for considering anti-TGFβ2 antibodies as a potential therapeutic approach to address multi-organ damage in the context of heart failure.

## Materials and Methods

### Animals

C57BL/6J mice (stock no. 000664) were from Jackson Laboratory. B-NDG mice were from Biocytogen (Beijing). *Cdh5*-CreERT2 mice (C001330) were from Cyagen (Guangzhou) and *Tgfbr2*-flox mice (NM-CKO-200026) from Shanghai Southern Model Biotechnology. Endothelial-specific *Tgfbr2* conditional knockout (cKO) mice were generated by crossing *Tgfbr2*-flox with *Cdh5*-CreERT2 mice, followed by tamoxifen induction to activate Cre recombinase before TAC surgery. All mice were on a C57BL/6J background, randomly assigned to groups, and housed under a 12-h light/dark cycle at 22 ± 2 °C with ad libitum access to food and water. All animal procedures were approved by the IACUC of Xiamen University and complied with ARVO guidelines.

### Culture of human endothelial cells

Human umbilical vein endothelial cells (HUVECs; Yizefeng Biotechnology, YPC-H001) and human primary retinal microvascular endothelial cells (HRMECs; Meisen Cell Technology, CTCC-150-HUM) were cultured in endothelial cell medium (ECM; ScienCell, 1001) supplemented with fetal bovine serum and growth factors at 37 °C in 5% CO_2_. HRMEC medium was additionally supplemented with 100 U/mL penicillin and 100 μg/mL streptomycin. Cells were detached with accutase (Invitrogen, 00-4555-56). For treatments, cells were seeded at 2 × 10^5^ cells per well in 24-well plates, cultured overnight for attachment, and then exposed to the indicated treatments for 48 h.

### Collection and processing of blood samples

Human peripheral blood was collected from 20 HF patients and 20 healthy controls at the Cardiovascular Hospital of Xiamen University with informed consent and ethics approval (No. XMH202449). HF diagnosis followed ACC/AHA criteria. Mouse blood was collected from the retroorbital sinus under isoflurane anesthesia into EDTA-containing tubes (Solarbio, YA1461). Human and mouse blood samples were centrifuged at 1,500 g for 10 min at 4 °C, and plasma was stored at -80 °C. Before use, plasma was dialyzed in saline using a 3.5 kDa D-tube dialyzer (EMD Millipore) at 4 °C with saline changes every 4 h over 48 h, sterilized by filtration through a 0.22 μm filter, and stored at -80 °C.

### Plasma treatment

HUVECs and HRMECs were seeded and allowed to attach for 24 h. Dialyzed human or mouse plasma was added to the culture medium at 1% (v/v) for 48 h, after which cells were harvested for senescence assays. For EV depletion experiments, plasma was first subjected to ultracentrifugation as described below.

### Extracellular vesicle depletion

To determine whether the senescence-inducing activity of HF plasma is associated with extracellular vesicles (EVs), plasma samples from normal/HF patients and Sham/TAC mice were subjected to EV depletion by differential ultracentrifugation. Briefly, plasma was centrifuged at 2,000 g for 20 min and then at 10,000 g for 30 min to remove residual debris and large vesicles. The resulting supernatant was subsequently ultracentrifuged at 100,000 g for 90 min at 4 °C to pellet small EVs. The EV-depleted supernatant was collected, diluted 1:100 in culture medium, and applied to endothelial cells for 48 h. Following treatment, cells were harvested for senescence-related assays.

### Establishment of the TAC model and cardiac function monitoring

Transverse aortic constriction was performed in 2-month-old C57BL/6J mice as previously described 25 to induce pressure-overload heart failure. Briefly, under isoflurane anesthesia, the aortic arch was exposed via thoracotomy and constricted between the innominate and left common carotid arteries using a 27-gauge needle and 6-0 silk suture. The needle was removed immediately after ligation. Sham-operated mice underwent thoracotomy without aortic constriction. All mice received postoperative antibiotics and analgesics. Cardiac function was monitored by echocardiography (Echo) at baseline and monthly thereafter, as described 25. LVEF%, LVFS%, LVID,d, LVID,s, LVEDV, and LVESV were measured.

### Retinal electroretinogram analysis

Retinal function was assessed by electroretinography (ERG) as previously described 14. Briefly, mice were dark-adapted for ≥ 12 h, anesthetized with isoflurane, and pupil-dilated with tropicamide. Scotopic ERG responses were recorded at stimulus intensities of 0.01–10 cd·s/m^2^ using gold-ring corneal electrodes. After 30 min of light adaptation (30 cd/m^2^ background), photopic responses were recorded at 3–100 cd·s/m^2^. Fifteen waveforms per animal were averaged. a-wave amplitude was measured from baseline to trough; b-wave amplitude was measured from the a-wave trough to the positive peak, or from baseline if no a-wave was present.

### Optical coherence tomography

Retinal structure was assessed by optical coherence tomography (4D-ISOCT; OPTOPROBE, UK). Retinal volumes were acquired from 200 vertical B-scans per eye (1,000 A-scans per B-scan). Retinal layer thickness was quantified using FIJI (NIH). Layers analyzed included GCL+IPL, IS+OS, and total retinal thickness.

### Parabiosis

Parabiosis was performed according to a previously established protocol 26. Briefly, 2-month-old WT mice were paired with age-matched Sham or TAC mice (surgery at 1.5 months, 2-week recovery). Under sodium pentobarbital anesthesia, skin incisions were made along the flanks, and partners were sutured at the elbow, knee, and dorsal-ventral skin with 4-0 silk.

### Primary microglia culture and synaptosome phagocytosis

Primary mouse microglia were isolated from mixed glial cultures prepared from P1–P3 mouse pups. Cultures were maintained in DMEM with 10% FBS, and microglial proliferation was promoted with GM-CSF (25 ng/mL; R&D Systems, 415-ML-050) added at day 3. Mature microglia were harvested by shaking (200 rpm, 30 min) at days 10–12. Synaptosomes were purified from mouse hippocampi and labeled with pHrodo Red (Thermo Fisher, P36600) as previously described 27. For the phagocytosis assay, microglia were seeded at 2 × 10^5^ cells per well, cultured for 24 h, treated as indicated, and incubated with pHrodo-labeled synaptosomes (1.2 μg/μL) for the final 3 h before immunocytochemical analysis.

### Histology and staining

Lectin staining. Enucleated eyes were fixed in 4% PFA for 1–2 h, and retinas were dissected, permeabilized in 0.2% Triton X-100 overnight at 4 °C, and incubated with fluorescent lectin (Thermo Fisher, I21413) overnight at 4 °C. Retinas were flat-mounted in four-leaf clover cuts and imaged on a Leica DMI4000B microscope. Sirius red staining. Frozen heart sections were fixed in 4% PFA, stained with 0.2% phosphomolybdic acid (2.5 min) followed by Picro-Sirius Red (Abcam, ab150681; 90 min), differentiated in 0.5% acetic acid, dehydrated, and coverslipped. Wheat germ agglutinin (WGA) staining. Frozen tissue sections were fixed in cold acetone (-20 °C, 10 min), incubated with 5 μg/mL WGA (Sigma, L4895) and DAPI (1 μg/mL; CST, 4083) for 30 min, and imaged on a Leica SP8 DLS confocal microscope.

### RNA extraction and qPCR analysis

Total RNA was extracted with TRIzol (Thermo Fisher, 15596026CN) and reverse-transcribed. qRT-PCR was performed with FastStart Universal SYBR Green Master (Roche, 04913850001) on a LightCycler 480II system (Roche). Target gene expression was calculated by the ΔΔCt method with β-actin as the internal control.

### RNA sequencing

RNA integrity was assessed on an Agilent 2100 Bioanalyzer. Libraries were prepared with the NEBNext Ultra RNA Library Prep Kit (NEB, E7530) and sequenced on an Illumina NovaSeq 6000 (150-bp paired-end). Raw reads were quality-checked with FastQC (v0.11.5), adapter-trimmed with Trim Galore (v3.4), and mapped to the human transcriptome (hg38, GENCODE v41) with STAR (v2.7.10b). Gene counts were obtained with featureCounts (v2.0.6) and differential expression was analyzed with DESeq2 (v1.42.0).

### Single-cell sequencing data and RNA-seq data analysis

Publicly available single-nucleus RNA-seq data (EGA: EGAS00001006374) and bulk RNA-seq data (GEO: GSE116250) from HF patients and healthy controls were analyzed. Single-nucleus data were processed with Seurat (v4.0): dimensionality reduction was performed by UMAP, differential expression was analyzed with DESeq2, and senescence scores were calculated with AUCell. GO enrichment was performed with clusterProfiler (v4.10.0), and heatmaps were generated with ComplexHeatmap.

### CUT&Tag

CUT&Tag was performed with the Hyperactive In-Situ ChIP Library Prep Kit (TD901, Vazyme). Paired-end reads were aligned with Bowtie2 (v2.5.1; -end-to-end -very-sensitive -no-mixed -no-discordant -I 10 -X 700). GO and motif enrichment analyses were performed with clusterProfiler.

### Co-immunoprecipitation

Endothelial cells were lysed in TNE buffer (20 mM Tris-HCl, pH 7.4; 100 mM NaCl; 1 mM EDTA; 0.5% NP-40) with protease inhibitors (Roche, 4693159001). Lysates were incubated with anti-SMAD2/3 (CST, 8685; 1:200) or normal rabbit IgG (CST, 2729) overnight at 4 °C, followed by incubation with Dynabeads Protein G (Thermo Fisher, 10004D) for 4 h. Bound proteins were eluted, resolved by SDS-PAGE, and immunoblotted with anti-EP300 (Santa Cruz, sc-48343; 1:1000).

### Chromatin immunoprecipitation

ChIP was performed as previously described 28 with modifications. Endothelial cells were cross-linked with 1% formaldehyde, quenched with 125 mM glycine, lysed, and sonicated to 150–350 bp fragments. Chromatin was incubated overnight at 4 °C with antibodies against SMAD2/3 (CST, 8685), EP300 (CST, 57625), or H3K9ac (CST, 9649), followed by incubation with Dynabeads Protein G for 4 h. After sequential washes and elution, DNA was purified and analyzed by qPCR on a LightCycler 480 system.

### RNA interference

siRNA transfections were performed with Lipofectamine RNAiMAX (Thermo Fisher, 13778100) according to the manufacturer's protocol. siRNAs targeting human SMAD2 (siSmad2-2) and EP300 (siEp300-3) and a non-targeting control (siN0000001-1-5) were from RiboBio. The siRNA sequences targeting human Smad2 and Ep300 were as follows:

si*Smad2*-2, sense: 5′-GUCCCAUGAAAAGACUUAA-3′; si*Smad2*-2, antisense: 5′-UUAAGUCUUUUCAUGGGAC-3′; si*Ep300*-3, sense: 5′-CAAUAGAGCGGAAUACUAU-3′; si*Ep300*-3, antisense: 5′-AUAGUAUUCCGCUCUAUUG-3′.

### Tail vein injections of TGFβ2

Recombinant TGFβ2 (6 ng per mouse in PBS) or PBS was injected via the tail vein every 2 days for 2 months, starting at 2 months of age. The dose was calculated to approximate the elevated plasma TGFβ2 concentration observed in HF patients (~6 ng/mL vs. ~1 ng/mL in healthy controls), assuming a total blood volume of ~2 mL per mouse. Mice were assessed 3 days after the final injection.

### Anti-TGFβ2 antibody administration

Intravitreal injection. TAC or Sham mice received six intravitreal injections of anti-TGFβ2 antibody (Invitrogen, MA5-37505; 1 mg/kg in 1 μL saline) or isotype control IgG (Abclonal, AC011) between 3 and 5 months of age. Injections were performed under isoflurane anesthesia using a 32 G needle under microscopic guidance. Mice were assessed at 5 months after a 1-week recovery. Tail vein injection. The same antibody and dosing schedule (1 mg/kg, six injections over 2 months) were administered via the tail vein for multi-organ senescence experiments. Cell treatment. Endothelial cells at 70–80% confluence were treated with anti-TGFβ2 antibody (1 μg/mL) or vehicle for 48 h before downstream analysis.

### ELISA

Plasma TGFβ2 was measured with a human TGFβ2 ELISA kit (Elabscience, E-EL-H1587) following the manufacturer's instructions. Plasma samples were acid-activated before assay to measure total TGFβ2. Absorbance was read at 450 nm, and TGFβ2 concentrations were calculated from a standard curve. Each sample was assayed in triplicate.

### Immunohistochemistry and immunocytochemistry

Immunohistochemistry (IHC). Tissue sections were prepared after perfusion fixation and stained as previously described 29. Primary antibodies: anti-Iba1 (Wako, 019-19741; 1:500), anti-CD31 (CST, 3528S; 1:100), and anti-β-galactosidase (CST, 2372S; 1:100). Confocal imaging was performed on a Leica SP8 DLS, and quantification used ImageJ (NIH). Immunocytochemistry (ICC). Cells were fixed in 4% PFA (30 min), permeabilized with 0.2% Triton X-100 (5 min), and blocked with 5% BSA (30 min). Primary antibodies (overnight, 4 °C): anti-Iba1 (Wako, 019-19741; 1:500; or SYSY, 234308; 1:500), anti-CD31 (CST, 3528S; 1:100), anti-p16INK4a (CST, 18769; 1:200), anti-β-galactosidase (CST, 2372S; 1:200), anti-p21 (CST, 2947S; 1:500), and anti-H3K9ac (CST, 5327S; 1:200). Secondary antibodies and DAPI were applied for 30 min at room temperature. Imaging and analysis used Leica SP8 DLS, ImageJ, and Imaris (Bitplane).

### Immunoblot analysis

Immunoblotting analysis was performed according to previously established protocols 30. Protein extracts were resolved by SDS-PAGE and transferred to PVDF membranes. Membranes were blocked with 5% non-fat dry milk (1 h, room temperature) and incubated overnight at 4 °C with primary antibodies: TGFβ2 (Proteintech, 19999-1-AP; 1:1000; or Invitrogen, MA5-37505; 1:1000), p-SMAD2 (CST, 3108; 1:1000), SMAD2 (CST, 3103; 1:1000), SMAD2/3 (CST, 8685; 1:1000), p-SMAD1/5/9 (CST, 13820; 1:1000), SMAD5 (CST, 12534; 1:1000), EP300 (Invitrogen, MA1-16608; 1:1000; or Santa Cruz, sc-48343; 1:1000), Histone H3 (CST, 14269; 1:1000), H3K9ac (CST, 9649; 1:1000), p21 (CST, 2947; 1:1000), and GAPDH (CST, 5174; 1:1000; or Rockland, 200-901-BJ5; 1:1000). HRP-conjugated secondary antibodies were applied for 1 h, and signals were detected by ECL (Meilunbio, MA0186). For mouse plasma, albumin was depleted with the Minute Albumin Depletion Kit (Invent Biotechnologies, WA-013). Bands were quantified with ImageJ and normalized to GAPDH.

### Flow cytometric sorting

Retinas were dissected and digested in HBSS containing 1.5 mg/mL collagenase II and 100 U/mL DNase I (37 °C, 15–20 min). For endothelial cell isolation, single-cell suspensions were stained with anti-CD31-APC and anti-CD45-FITC (1:200) and DAPI. Live CD31⁺CD45⁻ cells were sorted as retinal vascular endothelial cells. For microglia isolation, retinas were digested with 1 mg/mL collagenase D and 100 U/mL DNase I, stained with anti-CD11b-APC and anti-CD45-PE, and live CD11b^+^CD45^int^ cells were sorted as retinal microglia. Sorting was performed on a flow cytometer.

### Statistical analysis

All experiments were independently repeated at least thrice. Immunofluorescence data were analyzed using ImageJ software, whereas other experimental data were analyzed using GraphPad Prism software (version 8.0). Data are presented as mean ± standard error of the mean (SEM). Differences between the two groups were analyzed using a two-tailed Student’s *t*-test. One-way or two-way analysis of variance (ANOVA) was used for comparisons involving more than two groups. *P < 0.05; **P < 0.01; ***P < 0.001; ****P < 0.0001 were considered statistically significant.

## Results

### Heart failure induces retinal and vascular network damage

**Figure 1:**
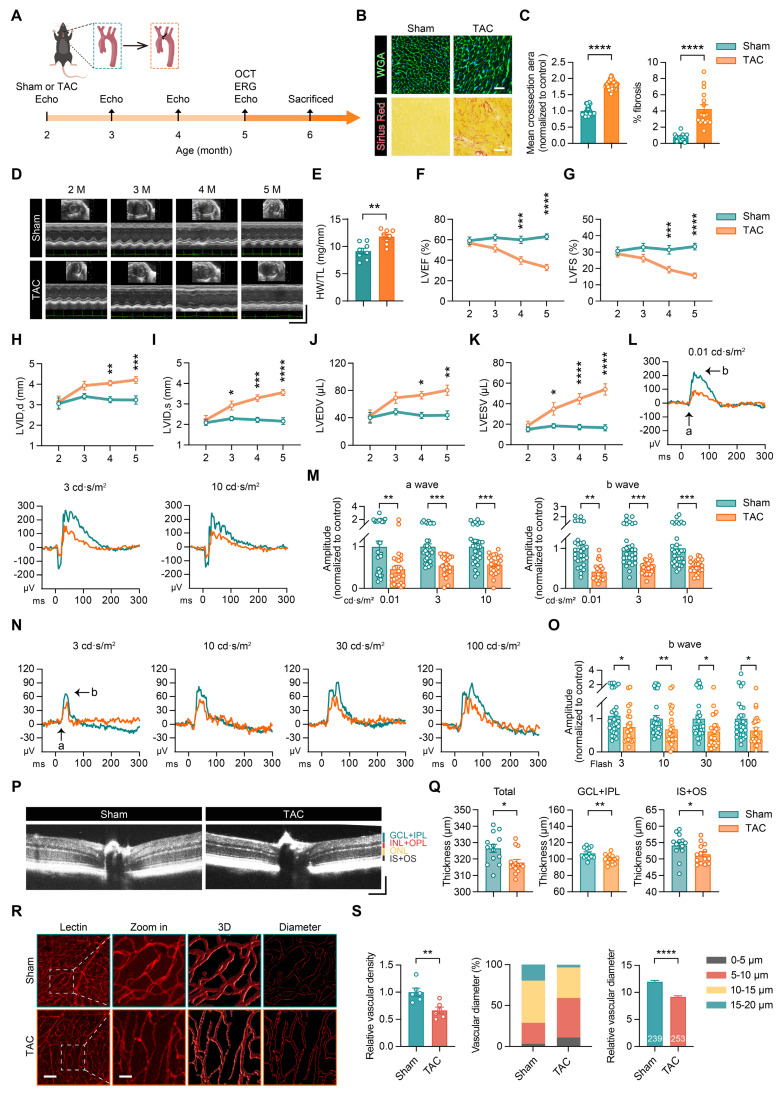
** Retinal and vascular networks were damaged in the HF mouse model.** (A) Schematic representation of the experimental timeline illustrating progression from Sham or TAC surgery to the end of the study. Surgeries were performed at 2 months of age using monthly echocardiographic assessments. OCT and ERG were conducted at five months of age, followed by tissue collection. (B-C) Representative histological images showing cross-sections of cardiomyocytes and fibrosis in the Sham and TAC groups, along with quantification of cardiomyocyte area and fibrosis percentage, n = 16 mice per group. Scale bar, 50 μm in the upper panels and 25 μm in the lower panels. (D) Echocardiographic images showing left ventricular function from 2 to 5 months of age in mice. Horizontal scale bar, 300 ms; vertical scale bar, 6 mm. (E) Heart weight (HW) to tibia length (TL) ratio at 5 months in Sham and TAC mice, n = 8 mice per group. (F-K) Temporal analysis of LVEF%, LVFS%, LVID,d, LVID,s, LVEDV, and LVESV in the Sham and TAC groups, n = 8 mice per group. (L-M) ERG waveform responses and quantification of a-wave and b-wave amplitudes under scotopic conditions in Sham and TAC mice, n = 24 to 30 eyes from 12 to 15 mice per group. (N-O) ERG b-wave responses and amplitude comparisons under photopic conditions between Sham and TAC mice, n = 24 to 28 eyes from 12 to 14 mice per group. (P-Q) OCT cross-sectional images of retinas from Sham and TAC mice, with quantifications of total retinal thickness, GCL+IPL, and IS+OS thicknesses, n = 13 to 14 mice per group. Horizontal scale bar, 130 μm; vertical scale bar, 150 μm. (R-S) Lectin-stained images showing retinal vasculature, including magnified views, 3D reconstructions, and projected vascular diameters, with quantification of retinal vascular density, the proportion of vessels with different diameters, and vessel diameter analysis, n = 239 to 253 vessels from 6 mice per group. Scale bar, 100 μm in the lectin images and 30 μm in the zoom-in images. All data are presented as the mean ± SEM. *P*-values were determined using Student’s t-test in (C), (E), (Q), and (S); repeated measures ANOVA with Bonferroni’s post hoc analysis in (F-K); and one-way ANOVA with Tukey’s post hoc analysis in (M) and (O). *p < 0.05, **p < 0.01, ***p < 0.001, ****p < 0.0001.

To assess the impact of HF on retina, we subjected 2-month-old mice to transverse aortic constriction (TAC) surgery and monitored their cardiac function monthly. At 5 months, retinal structure and function were evaluated using optical coherence tomography (OCT) and electroretinography (ERG) (Figure 1A). Compared to the sham-operated (Sham) group, the TAC group exhibited pronounced myocardial hypertrophy and fibrosis (Figures 1B-C). Echocardiographic analysis further demonstrated progressive left ventricular dysfunction, as evidenced by a decline in the left ventricular ejection fraction (LVEF%) and fractional shortening (LVFS%) (Figures 1D-G), as well as increases in HW/TL, the left ventricular internal diameter during diastole (LVID,d), systole (LVID,s), left ventricular end-diastolic volume (LVEDV) and end-systolic volume (LVESV) (Figures 1H-K). These findings confirmed the successful establishment of a HF model.

The ERG tests were conducted under both scotopic (dark-adapted) and photopic (light-adapted) conditions to evaluate retinal function. Under scotopic conditions, TAC mice showed reduced a-wave and b-wave amplitudes at light intensities of 0.01, 3, and 10 cd·s/m², indicating impaired rod photoreceptor function and bipolar cell signaling (Figures 1L-M). Similarly, under photopic conditions, TAC mice exhibited decreases in b-wave amplitudes at 3, 10, 30, and 100 cd·s/m², reflecting compromised cone photoreceptor and bipolar cell signaling function (Figures 1N-O).

OCT scans revealed a reduction in total retinal thickness in the TAC group, with notable thinning observed in the ganglion cell layer and inner plexiform layer (GCL+IPL), as well as in the inner and outer photoreceptor segments (IS+OS) (Figures 1P-Q). Furthermore, lectin staining highlighted a decrease in retinal vascular density and vessel diameter in TAC mice (Figures 1R-S), suggesting that heart failure not only disrupts retinal structure and function but also induces damage to the retinal vascular network.

### Heart failure plasma disrupts the retinal and vascular network

**Figure 2:**
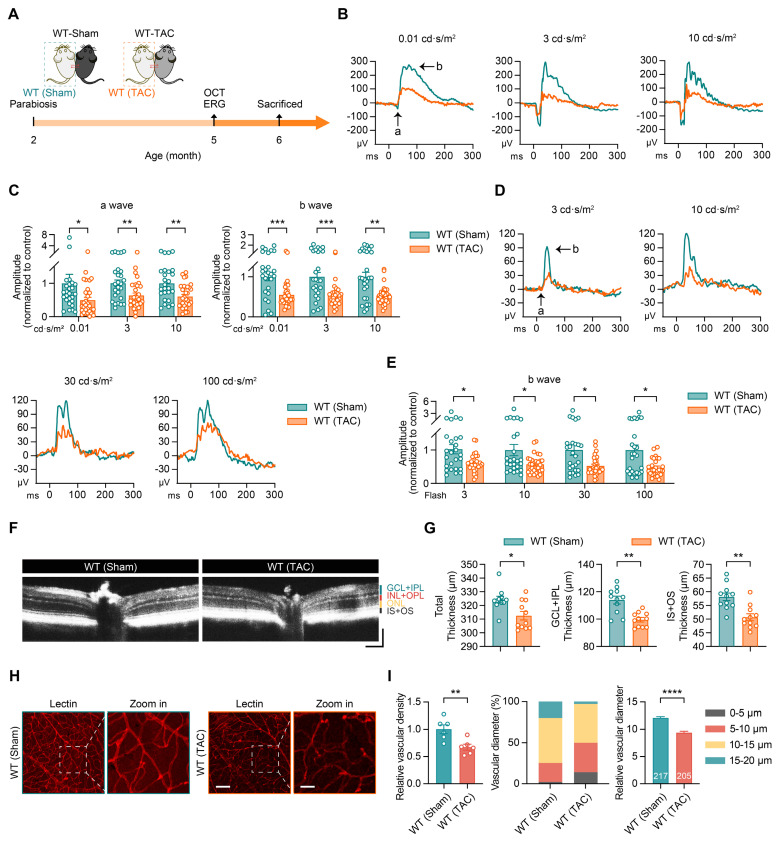
** Parabiosis with blood of HF mice leads to retinal and vascular damage.** (A) Schematic representation of experimental design. WT (Sham) refers to WT mice in parabiotic pairings with sham-operated mice and WT (TAC) refers to WT mice in parabiotic pairings with TAC-operated mice. (B-C) ERG waveform responses and quantification of a-wave and b-wave amplitudes under scotopic conditions in WT (sham) and WT (TAC) mice, n = 26 to 30 eyes from 13 to 15 mice per group. (D-E) ERG waveform responses and quantification of b-wave amplitudes under photopic conditions in WT (Sham) and WT (TAC) mice, n = 26 to 30 eyes from 13 to 15 mice per group. (F-G) OCT images of the retinas from WT (Sham) and WT (TAC) mice, with quantification of total retinal thickness, GCL+IPL, and IS+OS thicknesses, n = 11 mice per group. Horizontal scale bar, 130 μm; vertical scale bar, 150 μm. (H-I) Lectin-stained images showing retinal vascular networks in WT (Sham) and WT (TAC) mice, with quantification of vascular density, the proportion of vessels with different diameters, and vessel diameter analysis, n = 205 to 217 vessels from 6 mice per group. Scale bar, 100 μm in the lectin images and 30 μm in the zoom-in images. All data are presented as mean ± SEM. *P*-values were determined using one-way ANOVA with Tukey’s post hoc analysis in (C) and (E); Student’s t-test in (G) and (I). *p < 0.05, **p < 0.01, ***p < 0.001, ****p < 0.0001.

To explore the involvement of blood-borne factors in HF-induced retinal and vascular damage, we conducted a parabiosis experiment, allowing blood exchange between the two mice. This approach enabled us to evaluate the effect of HF plasma on the retinal structure and function of healthy wild-type (WT) mice. In this experiment, 2-month-old WT mice were paired with either the Sham or TAC mice. Retinal structure and function were assessed at 5 months of age using OCT and ERG (Figure 2A).

Our findings showed that exposure to HF plasma adversely affected retinal function in WT mice. Under dark-adapted conditions, the WT (TAC) group exhibited reduced a-wave and b-wave amplitudes, indicating impaired signal transduction function of rod photoreceptors and bipolar cells (Figures 2B-C). Similarly, under light-adapted conditions, the WT (TAC) group displayed lower b-wave amplitudes across different light intensities, reflecting diminished cone photoreceptor and bipolar cell activity (Figures 2D-E). Additionally, OCT scans showed a reduction in retinal thickness in the WT (TAC) group, with noticeable thinning in the GCL+IPL and IS+OS (Figures 2F-G). Lectin staining further revealed that HF plasma decreased retinal vascular density and reduced vessel diameter (Figures 2H-I). These observations indicate that components of HF plasma disrupt both the retinal structure and its vascular network through systemic circulation.

In summary, HF exerts systemic effects that extend beyond the heart and release circulating factors that contribute to retinal and vascular damage. These findings underscore the potential of HF to influence distant organs such as the retina via blood-mediated mechanisms.

### Heart failure plasma induces endothelial cell senescence

**Figure 3:**
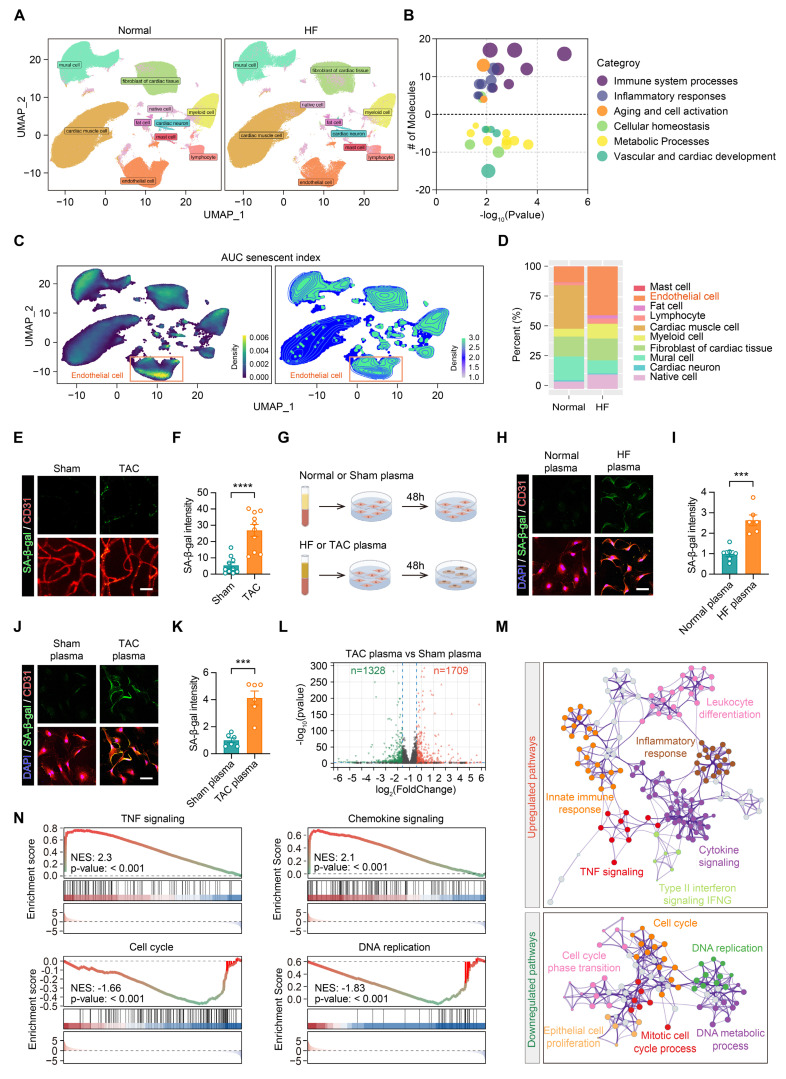
** Heart failure blood induces endothelial cell senescence.** (A) UMAP projection showing the distribution of cardiac cells in samples from normal and HF patients. The single-cell sequencing data was from the European Genome-Phenome Archive (accession number: EGAS00001006374). (B) GO enrichment analysis illustrating pathway enrichment of differentially expressed genes between HF patients and normal controls. Compared to normal controls, pathways above the dashed line are upregulated in heart failure patients, while pathways below the dashed line are downregulated. (C-D) UMAP projections displaying the AUC senescent index for each cell type in heart failure samples, with a comparison of the proportion of senescent cells between normal and heart failure samples. (E-F) Representative images and statistical analysis showing changes in SA-β-gal staining in retinal vascular endothelial cells from Sham and TAC mice, n = 10 mice per group. Scale bar, 35 μm. (G) Schematic diagram of the experimental design, showing treatment of endothelial cells with plasma from normal or heart failure patients, as well as plasma from Sham or TAC mice for 48 h. (H-I) Representative images and statistical analysis showing changes in SA-β-gal staining in endothelial cells after treatment with plasma from normal and heart failure patients, n = 6 independent experiments per group. Scale bar, 50 μm. (J-K) Representative images and statistical analysis showing changes in SA-β-gal staining in endothelial cells after treatment with plasma from Sham and TAC mice, n = 6 independent experiments per group. Scale bar, 50 μm. (L) Volcano plot showing differentially expressed genes in endothelial cells treated with plasma from Sham and TAC mice, n = 3 independent experiments per group. (M) Network diagram showing significantly upregulated and downregulated GO-enriched pathways in endothelial cells treated with plasma from the Sham and TAC mice. (N) Gene set enrichment analysis (GSEA) showing significant enrichment of TNF signaling, chemokine signaling, cell cycle, and DNA replication-related gene sets in endothelial cells treated with TAC plasma. All data are presented as mean ± SEM. *P*-values were determined using Student’s t-test in (F), (I), and (K). ***p < 0.001, ****p < 0.0001.

Following the confirmation that factors present in HF plasma contribute to retinal damage in mice, we sought to investigate the underlying molecular mechanisms. An analysis of single-nucleus RNA sequencing data (accession number: EGAS00001006374) from HF patient hearts revealed distinct differences in cell distribution compared with healthy controls (Figure 3A, Figure S1A-B). GO enrichment analysis indicated that pathways related to immune responses, inflammation, and cellular senescence were upregulated in patients with HF, while pathways involved in metabolic processes and vascular development were downregulated (Figure 3B). Further analysis using UMAP projections showed an elevated senescence score in endothelial cells from HF patients (Figures 3C-D). This suggests that endothelial cell senescence may impair vascular function and contribute to the progression of HF, with potential implications for distant organ systems.

We further assessed senescence-associated β-galactosidase (SA-β-gal) expression in retinal endothelial cells from the mice of the Sham and TAC groups. SA-β-gal levels were higher in the TAC group, indicating that HF promoted endothelial cell senescence in tissues beyond the heart (Figures 3E-F). To explore this further, human umbilical vein endothelial cells (HUVECs) were exposed to plasma from HF patients and TAC mice (Figure 3G). SA-β-gal expression increased in endothelial cells treated with HF patient plasma, while no such effect was observed in normal plasma treated cells (Figures 3H-I). Likewise, endothelial cells treated with plasma from TAC mice displayed elevated SA-β-gal levels compared to cells exposed to Sham plasma (Figures 3J-K), supporting the conclusion that HF plasma contains factors that induce endothelial cell senescence.

RNA sequencing of endothelial cells treated with TAC plasma revealed widespread alterations in gene expression (Figure 3L). GO enrichment network analysis identified upregulation in pathways related to tumor necrosis factor (TNF) signaling, chemokine signaling, innate immune responses, and inflammation, whereas pathways associated with the cell cycle and DNA replication were downregulated (Figure 3M). These findings were corroborated by Gene Set Enrichment Analysis (GSEA), which highlighted the enrichment of these inflammatory and senescence-related pathways in endothelial cells exposed to TAC plasma (Figure 3N). In conclusion, these data indicate that circulating factors in the HF plasma contribute to retinal and vascular damage by inducing endothelial cell senescence. Senescent endothelial cells activate immune and inflammatory pathways, reinforcing the SASP.

To further investigate whether the senescence-inducing activity of HF plasma is mediated by free soluble factors or extracellular vesicles (EVs), we depleted EVs from both normal/HF patient plasma and Sham/TAC mouse plasma by ultracentrifugation (100,000g, 90 min, 4°C) and treated endothelial cells with the EV-depleted supernatant for 48 h. EV-depleted HF plasma still significantly increased SA-β-gal expression in endothelial cells compared to EV-depleted normal plasma (Figure S1C-E). Similarly, EV-depleted TAC plasma induced higher SA-β-gal levels than EV-depleted Sham plasma (Figure S1F-G). These results indicate that the senescence-inducing factor, TGFβ2, exists predominantly as a free soluble protein in HF plasma rather than being encapsulated within extracellular vesicles.

### TGFβ2 mediates endothelial cell senescence and impairs retinal and vascular networks

**Figure 4:**
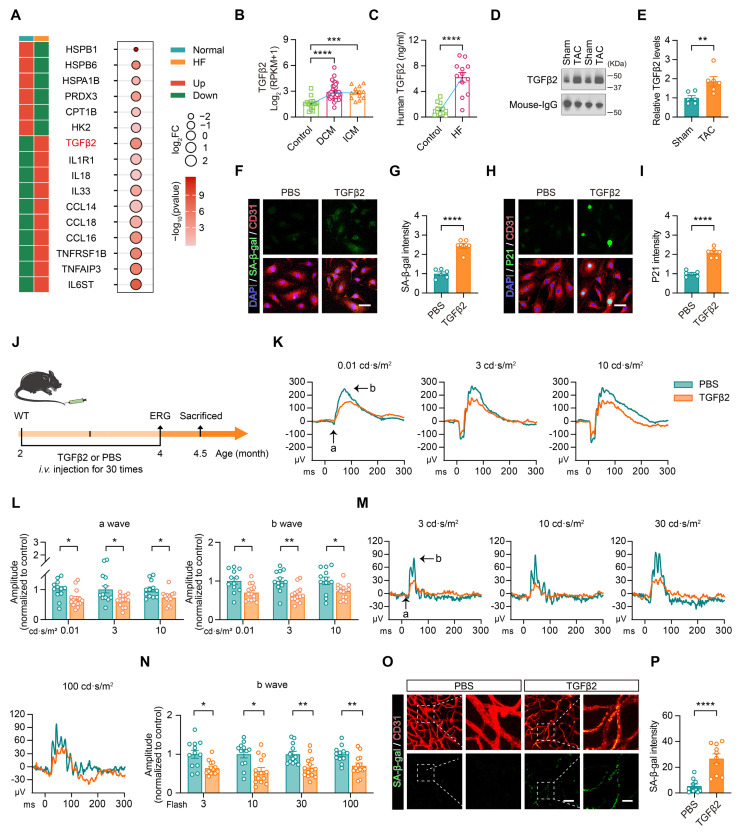
** Elevated TGFβ2 in heart failure blood induces endothelial cell senescence and retinal damage.** (A) Heatmap of RNA-seq data from GEO (accession number: GSE116250) showing differentially expressed genes significantly upregulated and downregulated in heart samples from HF patients compared to the normal controls. (B) RNA-Seq dataset GSE116250 showing TGFβ2 expression levels in normal, DCM, and ICM heart samples, n = 13 to 37 cases per group. (C) ELISA showing differences in plasma TGFβ2 concentrations between normal and heart failure patients, n = 12 cases per group. (D-E) Western blotting and quantification showing TGFβ2 protein levels in plasma from Sham and TAC mice, n = 6 mice per group. (F-G) Representative images and quantification of SA-β-gal staining in endothelial cells treated with PBS or TGFβ2 (6 ng/mL) for 48 h, n = 6 independent experiments per group. Scale bar, 50 μm. (H-I) Representative images and quantification showing changes in P21 expression levels in endothelial cells treated with PBS or TGFβ2 for 48 h, n = 6 independent experiments per group. Scale bar, 50 μm. (J) Schematic representation of the experimental timeline showing TGFβ2 protein injection into the tail vein of mice, starting at 2 months of age and administered every two days for two months. ERG was conducted at 4 months of age. (K-L) ERG waveform responses and quantification of a-wave and b-wave amplitudes under scotopic conditions in mice injected with PBS and TGFβ2 protein, n = 12 to 14 eyes from 6 to 7 mice per group. (M-N) ERG b-wave responses and amplitude comparisons under photopic conditions on mice injected with PBS and TGFβ2 protein, n = 12 to 14 eyes from 6 to 7 mice per group. (O-P) Representative images and quantification of changes in SA-β-gal staining in retinal vascular endothelial cells from PBS and TGFβ2 treated mice, n = 10 mice per group. Scale bar, 50 μm in the overview images and 15 μm in the zoom in images. All data are presented as mean ± SEM. *P*-values were determined by one-way ANOVA with Tukey’s post hoc analysis in (B), (L), and (N), and by Student’s t-test in (C), (E), (G), (I), and (P). *p < 0.05, **p < 0.01, ***p < 0.001, ****p < 0.0001.

To investigate the mechanisms by which HF plasma induces endothelial cell senescence, we conducted an in-depth analysis of single-nucleus RNA-seq data from HF patients. The analysis revealed an upregulation of genes related to inflammation in cardiac tissue from HF patients, which correlated with an increase in markers of the SASP, including TNF family cytokines and various interleukins, indicating their involvement in inflammatory responses and cellular senescence (Figure 4A). In parallel, TGFβ2 expression was notably elevated in the HF samples (Figure 4A). To identify the cardiac cellular origin of elevated TGFβ2, we further analyzed the single-nucleus RNA sequencing data from HF patients. UMAP visualization revealed a widespread increase in TGFβ2 expression across multiple cardiac cell populations in HF samples compared to normal controls (Figure S2A). Quantitative analysis demonstrated that TGFβ2 was significantly upregulated in cardiac fibroblasts, cardiac neurons, cardiomyocytes, and adipocytes (Figure S2B), indicating that the elevated circulating TGFβ2 in HF is derived from multiple cardiac cell types rather than a single cellular source. TGFβ2, a multifunctional cytokine involved in regulating cell proliferation, differentiation, and apoptosis, plays a key role in cardiac remodeling, fibrosis, and inflammation, all of which are characteristics of HF pathology 23, 24.

Further analysis of an additional RNA-Seq dataset (accession number: GSE116250) related to HF demonstrated that TGFβ2 levels were elevated in the blood of patients with dilated cardiomyopathy (DCM) and ischemic cardiomyopathy (ICM) compared to healthy controls (Figure 4B). Enzyme-linked immunosorbent assay (ELISA) confirmed that TGFβ2 concentrations in the plasma were higher in HF patients than in controls (Figures 4C), and this trend was also observed in TAC mice (Figures 4D-E).

To directly examine the role of TGFβ2 in inducing endothelial cell senescence, HUVECs were treated with TGFβ2. This treatment led to the upregulation of senescence markers, including SA-β-gal and P21, confirming that TGFβ2 promotes endothelial cell senescence (Figures 4F-I). To further evaluate the impact of TGFβ2 on retinal function, TGFβ2 protein was administered to mice via tail vein injection (Figure 4J). Under dark-adapted conditions, TGFβ2-treated mice exhibited reductions in both a- and b-wave amplitudes, reflecting impaired rod-bipolar cell signal transduction function (Figures 4K-L). Under light-adapted conditions, reduced b-wave amplitudes further indicated compromised cone-bipolar cell signal transduction function (Figures 4M-N). Furthermore, retinal endothelial cells from TGFβ2-treated mice exhibited higher levels of SA-β-gal expression than PBS-treated controls, providing additional evidence that TGFβ2 induces endothelial cell senescence (Figures 4O-P).

In summary, these findings demonstrated that elevated TGFβ2 levels in HF plasma contribute to endothelial cell senescence and impair retinal function, underscoring its role in HF-related retinal damage.

### TGFβ2 induces endothelial cell senescence via the pSMAD2/EP300-H3K9ac-P21 pathway

**Figure 5:**
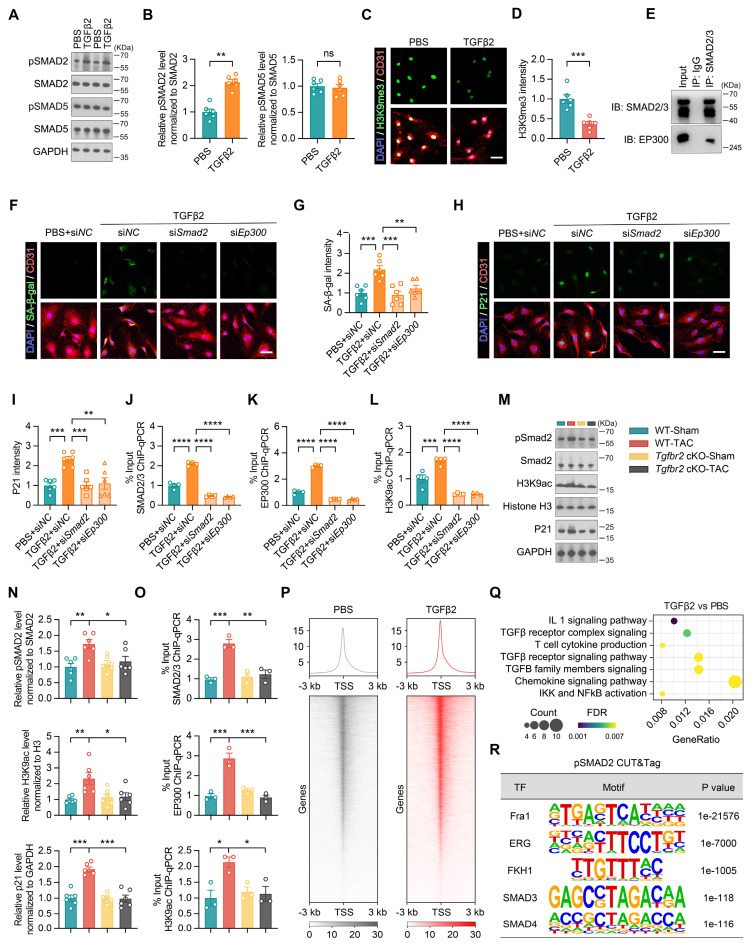
** TGFβ2 induces endothelial cell senescence by activating the pSMAD2/EP300-H3K9ac-P21 pathway.** (A-B) Western blotting and quantification showing changes in pSMAD2, SMAD2, pSMAD5, and SMAD5 protein levels in endothelial cells treated with PBS or TGFβ2, n = 6 independent experiments per group. (C-D) Representative images and quantification of H3K9me3 staining in endothelial cells after 48 h of treatment with PBS or TGFβ2, n = 6 independent experiments per group. Scale bar, 50 μm. (E) Co-IP showing the interaction between SMAD2/3 and EP300 in endothelial cells. (F-G) Representative images and quantification of SA-β-gal staining in endothelial cells treated with PBS or TGFβ2 (siNC, si*Smad2*, or si*Ep300*), n = 6 independent experiments per group. Scale bar, 50 μm. (H-I) Representative images and quantification of P21 staining in endothelial cells under different treatment conditions, n = 6 independent experiments per group. Scale bar, 50 μm. (J-L) ChIP-qPCR analysis showing enrichment of SMAD2/3, EP300, and H3K9ac at the first exon of the *Cdkn1a* (P21) gene in endothelial cells under different treatments, n = 4 independent experiments per group. (M-N) Western blot analysis and quantification showing changes in pSMAD2, SMAD2, H3K9ac, Histone H3, and P21 protein levels in flow cytometry-sorted retinal vascular endothelial cells from different experimental groups. 3 mice per group were pooled for each sample, n = 6 per group. (O) ChIP-qPCR analysis demonstrating the enrichment of SMAD2/3, EP300, and H3K9ac at the first exon of Cdkn1a in flow cytometry-sorted retinal vascular endothelial cells from different experimental groups. 3 mice per group were pooled for each sample; n = 6 per group. (P) CUT&Tag analysis showing pSMAD2 enrichment near the transcription start site (TSS) in endothelial cells, comparing PBS and TGFβ2 treatments, n = 3 independent experiments per group. (Q) GO enrichment analysis showing the significantly upregulated pathways following TGFβ2 treatment. (R) pSMAD2 CUT&Tag analysis showing the binding of Fra1, ERG, FKH1, SMAD3, and SMAD4 to pSMAD2 sites. All data are presented as the mean ± SEM. P-values were determined by Student’s t-test in (B) and (D), and by one-way ANOVA with Dunnett’s post hoc analysis in (G), (I-L), and (N-O). ns, not significant. *p < 0.05, **p < 0.01, ***p < 0.001, ****p < 0.0001.

The TGFβ signaling pathway is categorized into two distinct pathways: the classical SMAD-dependent pathway and the non-canonical SMAD-independent pathway 31, 32. In the SMAD-dependent pathway, TGFβ2 binds to its receptor, triggering the activation of the SMAD2 protein, which undergoes phosphorylation and subsequently translocates to the nucleus 31-33. TGFβ2 also activates SMAD5, which is typically associated with bone morphogenetic protein signaling 31-33. TGFβ2 specifically increased SMAD2 phosphorylation without influencing SMAD5, indicating that its primary effects were mediated through the pSMAD2 pathway (Figure 5A and 5B).

Aging is associated with widespread gene dysregulation, primarily driven by alterations in the chromatin structure 34-36. TGFβ2 treatment increased chromatin accessibility in endothelial cells, as indicated by the loss of the heterochromatin marker H3K9me3 (Figure 5C and 5D) 37, 38. As histone acetylation generally enhances chromatin accessibility and EP300 is a histone acetyltransferase involved in this process, we explored the interaction between pSMAD2 and EP300 38-40. Co-immunoprecipitation showed pSMAD2 binding EP300 (Figure 5E). Silencing *Smad2* or *Ep300* reduced SA-β-gal and P21 levels (Figure 5F through 5I). ChIP-qPCR confirmed that TGFβ2 enhanced SMAD2/3, EP300, and H3K9ac occupancy at the *Cdkn1a* (P21) locus, which was abrogated by either knockdown (Figure 5J through 5L).

To determine whether the enhanced TGFβ signaling in endothelial cells under HF conditions is driven by receptor upregulation, we examined TGFBR2 expression across multiple systems. RNA-seq analysis of HUVECs treated with TAC versus Sham plasma did not detect a significant change in *Tgfbr2* transcript levels (Table S1). This was further confirmed by qPCR in HUVECs treated with TAC mouse plasma or HF patient plasma, as well as in flow cytometry-sorted retinal vascular endothelial cells from TAC mice, all of which showed no significant alteration in *Tgfbr2* expression (Figure S3A-C). These results indicate that the pathological activation of TGFβ signaling in retinal endothelial cells is primarily driven by elevated circulating TGFβ2 ligand rather than by receptor upregulation. To further confirm the functional requirement of TGFBR2 in this signaling cascade, we generated endothelial-specific *Tgfbr2* conditional knockout (cKO) mice.

Western blot analysis of flow cytometry-sorted retinal vascular endothelial cells showed increased expression of pSMAD2, H3K9ac, and P21 in the WT-TAC group, whereas in the *Tgfbr2* cKO-TAC group, these proteins were restored to normal levels (Figure 5M and 5N). ChIP-qPCR analysis further confirmed that SMAD2/3, EP300, and H3K9ac enrichment in the first exon of Cdkn1a was elevated in the WT-TAC group but returned to normal levels in the *Tgfbr2* cKO-TAC group (Figure 5O). CUT & Tag demonstrated increased pSMAD2 peaks around the transcription start sites (Figure 5P). GO and motif analyses linked pSMAD2 targets to inflammatory, chemokine, and cell cycle arrest pathways (Figure 5Q and 5R). Collectively, TGFβ2 promotes endothelial epigenetic senescence by recruiting EP300 to acetylate H3K9 at senescence-associated genes; blockade of SMAD2 or EP300 prevents this cascade.

### Endothelial-specific *Tgfbr2* deletion protects against TAC-induced retinal injury

**Figure 6:**
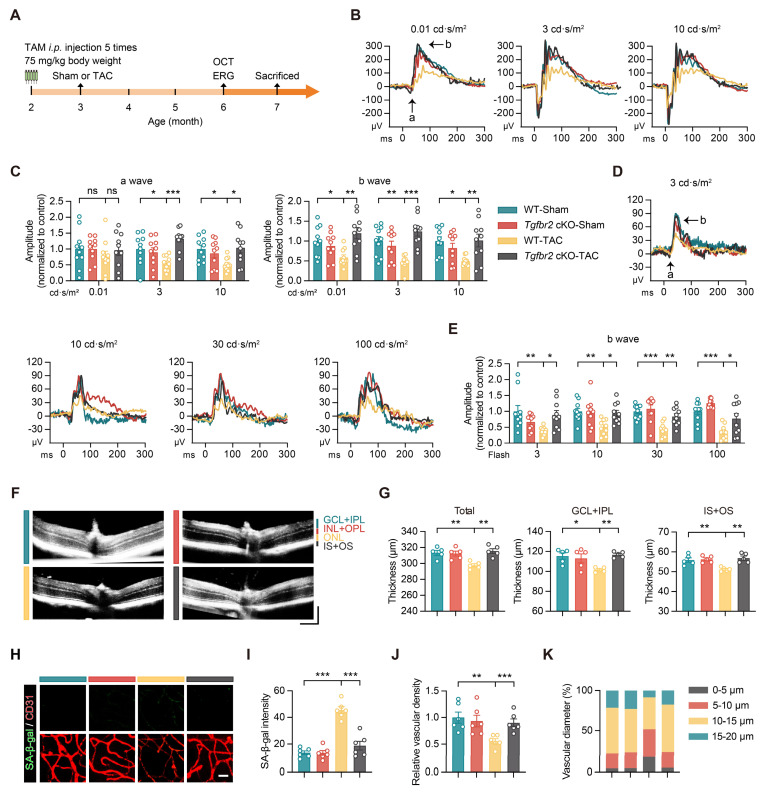
** Endothelial-specific *Tgfbr2* deletion protects against TAC-induced retinal dysfunction, vascular degeneration, and endothelial senescence.** (A) Schematic representation of the experimental timeline. Tamoxifen was administered by intraperitoneal injection before Sham or TAC surgery to induce endothelial-specific Tgfbr2 deletion. OCT and ERG were performed at 6 months of age, followed by tissue collection at 7 months of age. (B-C) Representative scotopic ERG waveforms and quantification of a-wave and b-wave amplitudes at flash intensities of 0.01, 3, and 10 cd·s/m^2^ in WT-Sham, *Tgfbr2* cKO-Sham, WT-TAC, and *Tgfbr2* cKO-TAC mice. (D-E) Representative photopic ERG waveforms and quantification of b-wave amplitudes at flash intensities of 3, 10, 30, and 100 cd·s/m^2^ across the indicated groups. (F-G) Representative OCT images and quantification of total retinal thickness, GCL+IPL thickness, and IS+OS thickness in the indicated groups. Horizontal scale bar, 130 μm; vertical scale bar, 150 μm. (H-I) Representative images and quantification of SA-β-gal staining in CD31^+^ retinal microvascular endothelial cells from the indicated groups. Scale bar, 35 μm. (J-K) Quantification of relative retinal vascular density and the distribution of retinal vascular diameters across the indicated groups. All data are presented as mean ± SEM. P-values were determined by one-way ANOVA with Tukey’s post hoc analysis in (C) and (E), and by one-way ANOVA with Dunnett’s post hoc analysis in (G), (I), and (J). ns, not significant. *p < 0.05, **p < 0.01, ***p < 0.001.

To determine whether endothelial *Tgfbr2* mediates TAC-induced retinal injury *in vivo*, we generated endothelial-specific *Tgfbr2* conditional knockout mice by crossing *Tgfbr2*-flox mice with *Cdh5*-CreERT2 mice followed by tamoxifen induction (Figure 6A). Quantitative PCR analysis of flow cytometry-sorted retinal microvascular endothelial cells confirmed efficient deletion of *Tgfbr2* in *Tgfbr2* cKO mice (Figure S4A).

We next assessed the effect of endothelial-specific *Tgfbr2* deletion on retinal structure and function after TAC. Under scotopic conditions, TAC-induced reductions in both a-wave and b-wave amplitudes were markedly attenuated in *Tgfbr2* cKO mice (Figure 6B-C). Similarly, under photopic conditions, the decrease in b-wave amplitudes observed in WT-TAC mice was significantly rescued by endothelial-specific *Tgfbr2* deletion (Figure 6D-E). OCT analysis further showed that *Tgfbr2* cKO preserved total retinal thickness as well as GCL+IPL and IS+OS thicknesses in TAC mice (Figure 6F-G). In parallel, SA-β-gal staining revealed that TAC-induced endothelial senescence in retinal microvascular endothelial cells was markedly reduced in *Tgfbr2* cKO mice (Figure 6H-I). Moreover, endothelial-specific *Tgfbr2* deletion restored retinal vascular density and improved vascular diameter distribution in TAC mice (Figure 6J-K).

To further determine whether these protective effects were secondary to alterations in the overall cardiac phenotype, we evaluated cardiac structure and function in WT and *Tgfbr2* cKO mice after TAC. Although *Tgfbr2* deletion efficiently reduced endothelial *Tgfbr2* expression, it did not significantly rescue TAC-induced cardiac hypertrophy or systolic dysfunction, as reflected by HW/TL, LVEF, LVFS, LVID,d, LVID,s, LVEDV, and LVESV (Figure S4B-I). These findings suggest that the retinal protection conferred by endothelial-specific *Tgfbr2* deletion is unlikely to be merely secondary to improved cardiac performance, but rather reflects a direct role of endothelial *Tgfbr2* signaling in mediating the cardio-retinal pathogenic axis.

### Endothelial cell senescence-induced microglial activation leads to retinal neuronal damage

The retina, as part of the central nervous system, is protected by the blood-retinal barrier, which prevents the entry of circulating factors, such as TGFβ2 41. We hypothesized that elevated levels of TGFβ2 in the bloodstream of patients with HF may induce endothelial cell senescence, leading to the release of SASP factors that alter the retinal microenvironment. Microglia, the primary immune cells within the central nervous system, are highly responsive to inflammatory and immune signals 42. Once activated, microglia exhibit enhanced phagocytic activity and initiate inflammatory cascades, which results in retinal neuronal damage.

**Figure 7:**
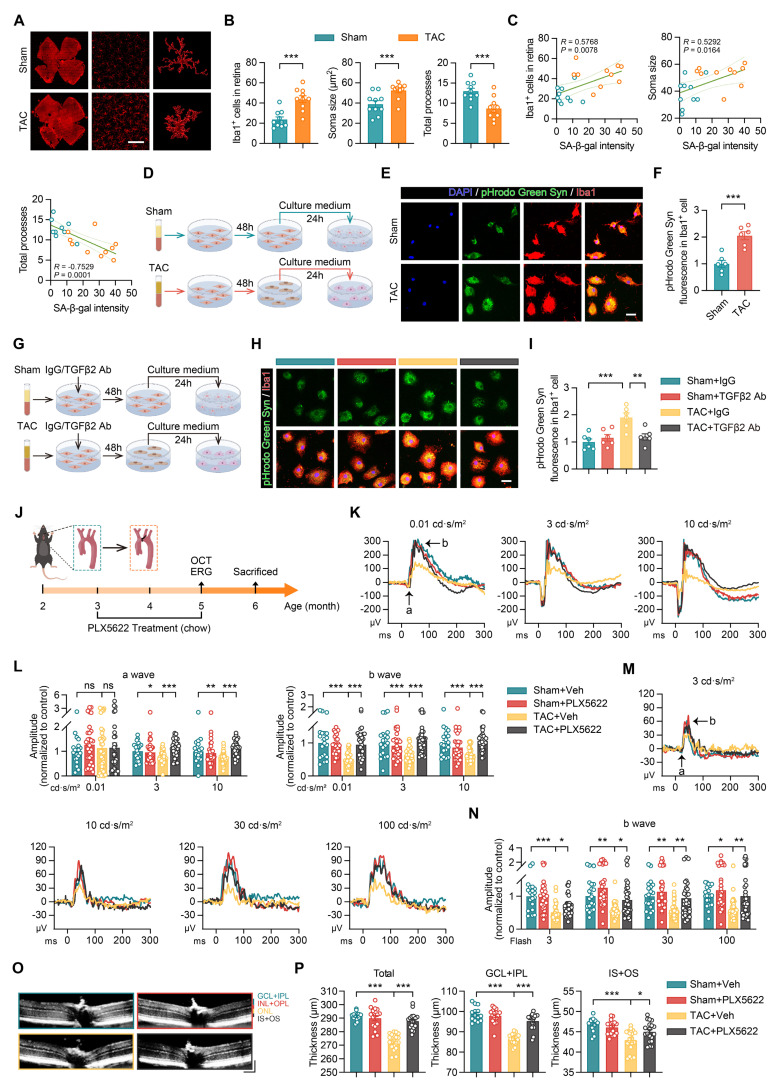
** Activation of microglia by senescent endothelial cells leads to retinal structural and functional damage.** (A-B) Representative images and quantitative analysis showing the number of Iba1^+^ cells, cell area, and total number of processes in the retinas of Sham and TAC mice, n = 10 mice per group. Scale bar, 150 μm. (C) Correlation between the number of Iba1^+^ cells, soma area, total number of processes, and SA-β-gal intensity in retinal endothelial cells (Figure 3E-F), n = 10 mice per group. (D) Schematic representation of the experimental timeline. (E-F) Representative images and quantitative analysis showing the fluorescence intensity of pHrodo Green Syn in Iba1^+^ microglia after plasma treatment in the Sham and TAC groups, n = 6 independent experiments per group. Scale bar, 25 μm. (G) Schematic representation of the experimental timeline. (H-I) Representative images and quantitative analysis showing changes in the fluorescence intensity of pHrodo Green Syn in Iba1^+^ microglia and the fluorescence intensity of Iba1^+^ cells in different treatment groups, n = 6 independent experiments per group. Scale bar, 25 μm. (J) Schematic showing the experimental timeline for Sham or TAC surgery performed on 2-month-old WT mice, followed by PLX5622 treatment (1.2 g/kg, provided ad libitum) from 3 to 5 months. OCT and ERG measurements were performed at 5 months, and tissue analysis was conducted at 6 months of age. (K-L) Scotopic ERG responses showing waveform plots and quantitative analysis of a-wave and b-wave amplitudes across groups, n = 20 to 40 eyes from 10 to 20 mice per group. (M-N) Photopic ERG responses showing waveform plots and quantitative analysis of b-wave amplitudes across groups, n = 20 to 40 eyes from 10 to 20 mice per group. (O-P) OCT cross-sectional images showing retinal structural changes across groups, with quantitative analysis, n = 14 to 20 mice per group. Horizontal scale bar, 130 μm; vertical scale bar, 150 μm. All data are presented as mean ± SEM. *P*-values were determined using Student’s t-test in (B) and (F), one-way ANOVA with Dunnett’s post hoc analysis in (I) and (P), and one-way ANOVA with Tukey’s post hoc analysis in (L) and (N). ns, not significant. *p < 0.05, **p < 0.01, ***p < 0.001.

Immunofluorescence staining showed an increase in both the number and soma size of Iba1^+^ microglia in the retinas of TAC mice, accompanied by a decrease in the number of total processes, indicating a transition to an activated state (Figures 7A-B). Further analysis identified a correlation between the level of microglial activation and SA-β-gal expression in retinal vascular endothelial cells, suggesting that microglial activation is associated with endothelial cell senescence (Figure 7C).

To determine whether microglial activation was mediated by SASP factors, endothelial cells were incubated with plasma from the Sham and TAC groups for 48 h. 4 hours prior to the conclusion of this incubation, the medium was replaced with a microglia-specific medium to allow conditioning with endothelial cell-derived factors. The conditioned media was then collected and applied to microglial cultures for an additional 24-hour incubation. During the final 3 h, microglia were exposed to pHrodo green-labelled synaptosomes to assess their phagocytic activity. Microglia treated with conditioned media from endothelial cells exposed to TAC plasma showed an increase in phagocytic function, as evidenced by the elevated pHrodo green fluorescence intensity (Figures 7D-F). This increase was diminished following the addition of an anti-TGFβ2 antibody (Figures 7G-I). Furthermore, quantitative PCR analysis demonstrated higher levels of inflammatory markers, including IL-6, TNF-α, IL-1β, and IFN-γ, in microglia treated with conditioned medium from TAC plasma-exposed endothelial cells (Figure S3D). The addition of anti-TGFβ2 antibodies reduced the expression of these proinflammatory factors (Figure S3E).

These findings indicate that TGFβ2 in the circulation of patients with HF induces endothelial cell senescence, which promotes the release of SASP factors. These factors activate microglia, leading to increased phagocytic activity and the initiation of inflammatory processes, ultimately contributing to retinal neuronal damage.

### Microglial depletion mitigates retinal structural and functional damage in heart failure

To investigate the role of microglial activation in heart failure-induced retinal damage, we administered PLX5622, a small molecule inhibitor of colony-stimulating factor-1 receptor, to eliminate microglia in Sham or TAC mice (Figure 7J). In dark-adapted ERG tests, TAC mice showed reduced a-wave and b-wave amplitudes, indicating impaired retinal function (Figures 7K-L). After PLX5622 treatment, the ERG responses in TAC mice showed improvement (Figures 7K-L). Under light-adapted conditions, PLX5622 treatment also increased b-wave amplitudes across different light intensities, indicating a protective effect on retinal function (Figures 7M-N). OCT revealed that TAC mice exhibited reduced total retinal thickness, GCL+IPL thickness, and photoreceptor layer thickness, reflecting structural damage to the retina (Figures 7O-P). Treatment with PLX5622 restored these retinal layers, further indicating the involvement of microglia in the structural damage observed in heart failure (Figures 7O-P). In conclusion, this study demonstrates that endothelial cell senescence triggered by HF contributes to both structural and functional retinal damage mediated by microglial activation. Microglial depletion using PLX5622 interrupts this pathological process, leading to improved retinal integrity and function.

### Anti-TGFβ2 antibody reduces endothelial cell senescence induced by HF plasma

**Figure 8:**
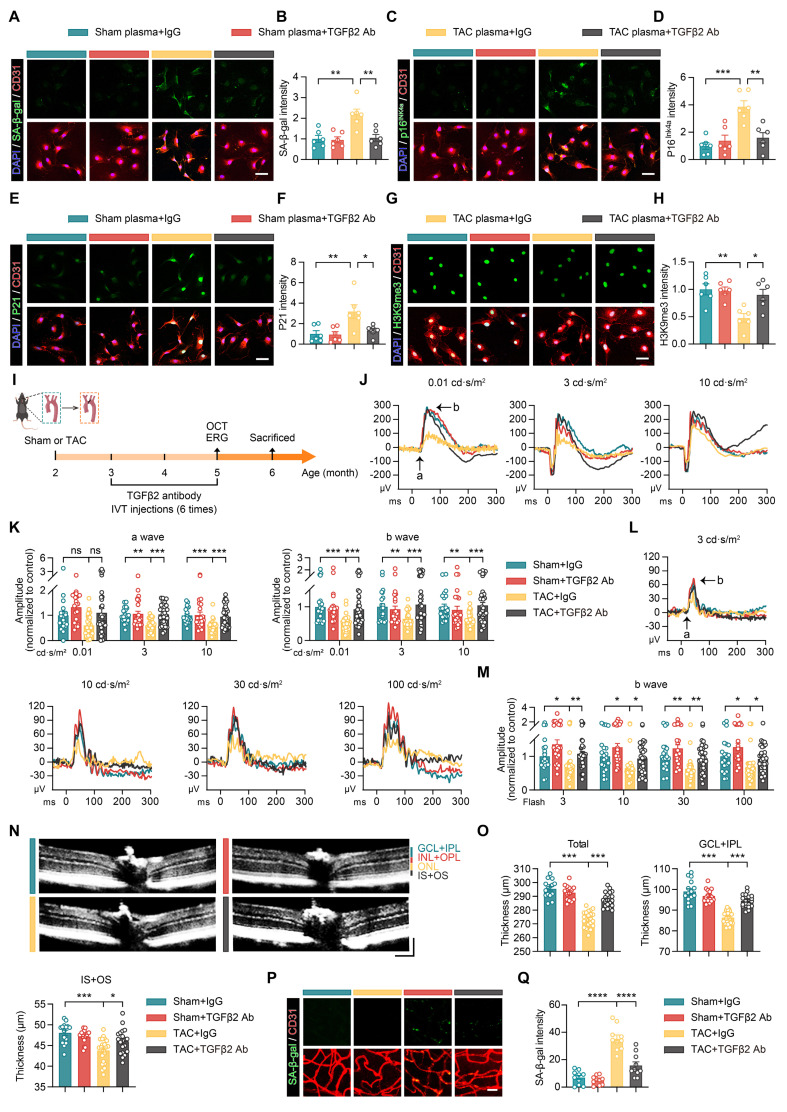
** Anti-TGFβ2 antibody treatment improves retinal and vascular network damage in a mouse model of heart failure.** (A-H) Representative images and quantitative analysis showing the results after treating endothelial cells with Sham or TAC plasma and co-treatment with IgG or anti-TGFβ2 antibody for 48 h. The expression levels of SA-β-gal (A-B), P16^Ink4a^ (C-D), P21 (E-F), and H3K9me3 (G-H) were assessed, n = 6 independent experiments per group. Scale bar, 50 μm. (I) Schematic of the experimental timeline. Sham or TAC surgery was performed on 2-month-old WT mice, followed by a 1-month recovery period. From 3 to 5 months of age, the mice received 6 intravitreal injections of the anti-TGFβ2 antibody. OCT and ERG assessments were conducted at 5 months of age, followed by histological analysis at 6 months of age. (J-K) Scotopic ERG responses showing ERG waveforms and quantitative analysis of a-wave and b-wave amplitudes in each group, n = 20 to 34 eyes from 10 to 17 mice per group. (L-M) Photopic ERG responses showing ERG waveforms and quantitative analysis of b-wave amplitudes in each group, n = 20 to 34 eyes from 10 to 17 mice per group. (N-O) OCT cross-sectional images showing structural changes in the retina across groups, with quantitative analysis, n = 15 to 22 mice per group. Horizontal scale bar, 130 μm; vertical scale bar, 150 μm. (P-Q) Representative images and quantitative analysis of SA-β-gal expression in retinal vascular endothelial cells across groups, n = 10 mice per group. Scale bar, 35 μm. All data are presented as mean ± SEM. *P*-values were determined by one-way ANOVA with Dunnett’s post hoc analysis in (B), (D), (F), (H), (O), and (Q) and by one-way ANOVA with Tukey’s post hoc analysis in (K) and (M). ns, not significant. *p < 0.05, **p < 0.01, ***p < 0.001, ****p < 0.0001.

Our findings indicate that elevated TGFβ2 levels in the plasma of patients with HF promote endothelial cell senescence by activating the pSMAD2/EP300-H3K9ac-P21 signaling pathway. To further delineate the role of TGFβ2 in this process and assess the effect of an anti-TGFβ2 antibody on endothelial cell senescence, we treated endothelial cells with plasma from both Sham and TAC mice and co-administered either IgG control or anti-TGFβ2 antibody. After 48 h of treatment, analysis of senescence-associated markers demonstrated that TAC plasma increased the expression of SA-β-gal, P16^Ink4a^, and P21 in endothelial cells, while anti-TGFβ2 antibody treatment effectively reduced these markers (Figures 8A-F). Additionally, the decline in H3K9me3 expression observed in TAC plasma-treated cells was mitigated by the anti-TGFβ2 antibody (Figures 8G-H).

To expand our understanding of the impact of the anti-TGFβ2 antibody on gene expression in endothelial cells, RNA-seq analysis was conducted. Principal component analysis (PCA) demonstrated that antibody treatment modified the gene expression profile altered by TAC plasma (Figure S5A). GO enrichment analysis indicated that antibody treatment enhanced pathways associated with DNA repair, cell cycle regulation, and DNA damage response, while downregulating pathways linked to cellular senescence, such as stress response, TP53 signaling, and oxidative stress response (Figure S5B). These findings suggest that the anti-TGFβ2 antibody effectively reduces endothelial cell senescence induced by TAC plasma.

### Intravitreal injections of the anti-TGFβ2 antibody mitigate retinal and vascular network damage in HF mice

To assess the therapeutic potential of anti-TGFβ2 antibody on retinal and vascular damage induced by HF, we performed Sham or TAC surgery in 2-month-old mice. After a 1-month recovery period, from months 3 to 5, the mice received 6 intravitreal injections of anti-TGFβ2 antibody. OCT and ERG were performed at 5 months of age (Figure 8I). ERG results showed that the TAC group exhibited reduced a- and b-wave amplitudes across various light intensities under dark-adapted conditions (Figure 8J-K). Treatment with anti-TGFβ2 antibody improved these amplitudes (Figure 8J-K). Similarly, under light-adapted conditions, b-wave amplitudes in the TAC group were reduced at different light intensities, and these amplitudes improved following anti-TGFβ2 antibody treatment (Figure 8L-M), indicating restoration of retinal function.

OCT scans further revealed that the TAC group had a reduced total retinal thickness, with thinning observed in the GCL+IPL and the IS+OS. Treatment with the anti-TGFβ2 antibody restored these retinal layer thicknesses (Figure 8N-O). Additionally, SA-β-gal staining indicated an increased expression of SA-β-gal in retinal vascular endothelial cells in the TAC group (Figure 8P-Q). This expression was reduced following treatment with the anti-TGFβ2 antibody, suggesting that the antibody effectively mitigated retinal vascular endothelial cell senescence (Figure 8P-Q).

In conclusion, our findings demonstrate that anti-TGFβ2 antibodies reduce HF-induced retinal and vascular damage. In vitro experiments showed that the antibody attenuated endothelial cell senescence caused by HF plasma by inhibiting the pSMAD2/EP300-H3K9ac-P21 pathway and normalizing associated gene expression. In vivo, intravitreal injection of anti-TGFβ2 antibody reduced endothelial cell senescence and improved retinal structure and function in HF mice. These results support the potential clinical application of the anti-TGFβ2 antibody in preventing and treating retinal damage associated with heart failure.

### Anti-TGFβ2 antibody reduces endothelial cell senescence in multiple organs of heart failure mice

**Figure 9:**
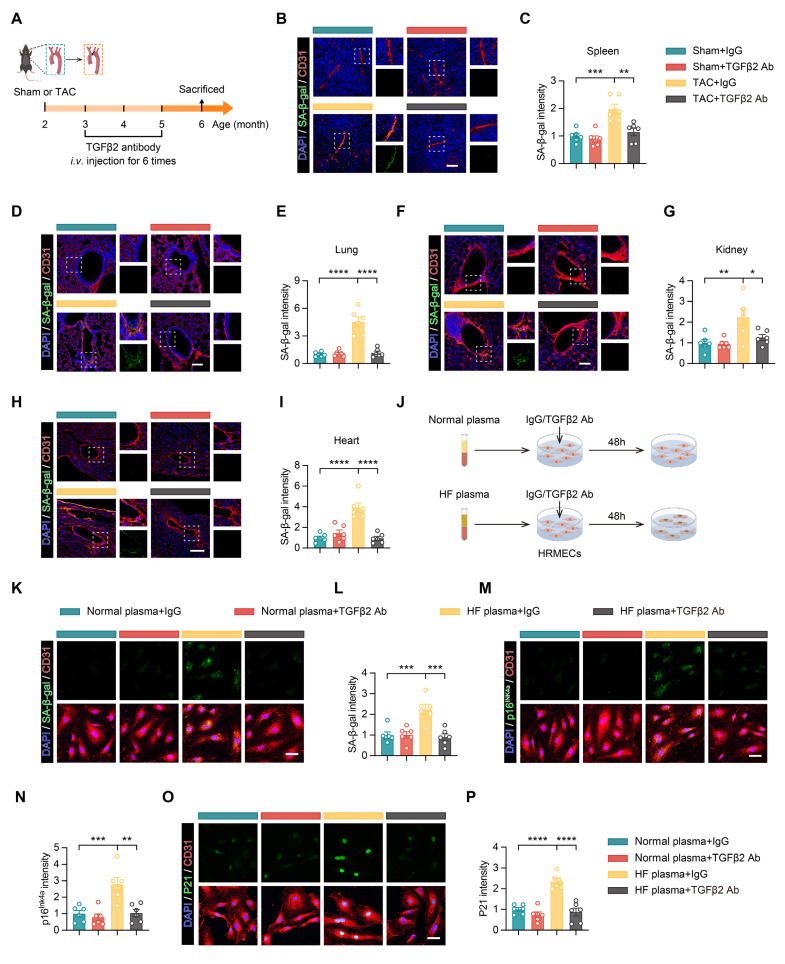
** TGFβ2 antibody ameliorates multi-organ endothelial cell senescence caused by HF and prevents HRMECs senescence induced by HF patient plasma.** (A) Schematic timeline showing the administration of the TGFβ2 antibody from 2 to 5 months of age in Sham and TAC mice. (B-I) Representative immunofluorescence images showing the intensity of SA-β-gal staining in CD31-labeled endothelial cells from the spleen, lungs, kidneys, and heart, n = 6 mice per group. Scale bar, 100 μm. (J) Experimental workflow diagram. (K-P) Representative images and quantitative analyses of HRMECs treated with normal or HF plasma, along with IgG or TGFβ2 antibody for 48 h. The expression levels of SA-β-gal (K-L), P16^Ink4a^ (M-N), and P21 (O-P) were measured in the endothelial cells, n = 6 independent experiments per group. Scale bar, 50 μm. All data are presented as mean ± SEM. *P*-values were determined by one-way ANOVA with Dunnett’s post hoc analysis for (C), (E), (G), (I), (L), (N), and (P). *p < 0.05, **p < 0.01, ***p < 0.001, ****p < 0.0001.

To assess the impact of the anti-TGFβ2 antibody on endothelial cell senescence across multiple organs induced by HF, we conducted Sham or TAC surgery on 2-month-old mice. 1 month after surgery, anti-TGFβ2 antibody treatment was initiated via tail vein injection and administered at regular intervals until the mice reached 5 months of age, with a total of 6 injections (Figure 9A). Immunofluorescence staining was used to examine the expression levels of the senescence marker SA-β-gal in endothelial cells from the spleen, lungs, kidneys, and heart of TAC mice. Compared with the Sham group, the TAC surgery led to an increase in SA-β-gal levels in endothelial cells, indicating accelerated senescence in these organs due to HF (Figures 9B-I). In contrast, the group treated with the anti-TGFβ2 antibody showed reduced SA-β-gal expression across all examined organs, with levels comparable to those observed in the Sham group (Figures 9B-I). These findings suggest that anti-TGFβ2 antibody administration reduces endothelial cell senescence induced by TAC across multiple organs, including the spleen, lungs, kidneys, and heart, demonstrating its potential protective role against HF-related systemic endothelial damage.

### Anti-TGFβ2 antibody mitigates senescence of primary human retinal microvascular endothelial cells induced by plasma from HF patients

To explore the role of TGFβ2 in human retinal microvascular endothelial cell senescence and to evaluate the potential therapeutic effect of an anti-TGFβ2 antibody, we selected primary human retinal microvascular endothelial cells (HRMECs) for experiments. This model was chosen to closely replicate physiological conditions in humans. HRMECs were treated with plasma derived from either healthy individuals or HF patients for 48 h. Simultaneously, an IgG control or anti-TGFβ2 antibody was administered to investigate its ability to inhibit senescence induced by HF plasma (Figure 9J). The results indicated that plasma from HF patients, compared to that from healthy controls, elevated the expression of SA-β-gal, P16^Ink4a^, and P21 in HRMECs, suggesting accelerated cellular senescence (Figures 9K-P). The anti-TGFβ2 antibody reduced the expression of these senescence markers, demonstrating its inhibitory effect on endothelial cell senescence triggered by HF plasma (Figures 9K-P).

In conclusion, this study illustrates that the anti-TGFβ2 antibody confers a protective effect against multi-organ endothelial cell senescence associated with HF. Specifically, in HRMECs, the antibody reduced senescence induced by HF plasma. These findings underscore the potential clinical relevance of targeting TGFβ2 to prevent or treat retinal microvascular damage in HF, highlighting its therapeutic potential.

## Discussion

Our research indicated that TGFβ2 has a new but detrimental role in HF-associated retinal damages. Our results showed that plasma from HF mice damaged the retina and vascular tissues of healthy mice, indicating that HF has systemic effects on distant organs via blood. Under HF conditions, TGFβ2 levels were found to be high which induced endothelial cell epigenetic senescence via the pSMAD2/EP300-H3K9ac-P21 signalling. SASP factor release activates retinal microglia in senescence, enhancing phagocytosis, causing neuroinflammatory response, and retinal neuronal damage. Earlier studies have established the importance of TGFβ2 in inflammation and tissue fibrosis. However, ours is the first to discover its major role in the cardio-retinal axis.

Based on clinical studies that have been done so far, HF has an impact on the diameter of the retinal vessels likely related to systemic inflammation and metabolic disturbance. However, most are observational with a lack of clear mechanistic insight 15, 17-21. Our study was aimed at clarifying how the TGFβ2 contributes to HF-induced retinal pathology, with a specific emphasis on the TGFβ2-mediated epigenetic regulation of endothelial cell senescence. This fills an important gap in our knowledge of HF-related retinal dysfunction along with the concept of the cardio-retinal axis. The retina, contributing to the central nervous system, works closely with brain activity and cardiac health 15. Hypertensive retinal damage is caused by hemodynamic and metabolic imbalance but also by the direct damage to retinal vascular endothelial cells by circulating factors like TGFβ2. This mechanism presents a novel explanation for the impact of HF on the retina.

To increase the translational value of our data, we validated our findings using primary human retinal microvascular endothelial cells treated with plasma from patients with heart failure. The similarity of the study system to human physiological conditions increased the utility of our findings. According to the evidence, treatment with anti-TGFβ2 antibody reduced the expression of senescence markers. This indicates that anti-TGFβ2 antibody can mitigate pathogenic factors in human heart failure plasma that induce endothelial cell senescence. The validated approach in human cells and patient samples bolsters the clinical potential to target TGFβ2 to prevent or treat retinal microvascular damage associated with heart failure. The insights into mechanism aforementioned from our mouse models and support for the clinical application of anti-TGFβ2 approach findings are consistent with the.

Significantly, TAC-induced retinal dysfunction, vascular degeneration, and endothelial senescence were greatly attenuated by endothelial-specific *Tgfbr2* deletion, although global cardiac remodelling and systolic function were not significantly improved. This separation supports our main conclusion that the cardio-retinal axis observed in this study represents an actively driven inter organ pathogenic pathway driven by the circulation of TGFβ2 rather than a passive peripheral result of cardiac dysfunction. As represented in Figure 9, our results show that the systemic neutralization of TGFβ2 attenuated cardiac endothelial cell senescence confirming the role of circulating TGFβ2 in cardiac endothelial injury. Nevertheless, findings indicate that endothelial senescence is not the primary driver of cardiac dysfunction in the TAC model, thus blockade of this pathway alone is not enough to achieve significant recovery of overall cardiac function. As the main site of disease, the heart experiences prolonged mechanical pressure overload. The pressure overload itself directly drives myocardial hypertrophy, fibrotic remodeling, and cardiomyocyte injury. Meanwhile, blockade of endothelial TGFβ2 signaling does not erase this mechanical stress. In contrast, the retina being a distal target organ gets damaged mostly by circulating TGFβ2 and the blockade of this signal is sufficient to effectively break the local pathological cascade. The above observations open up possibilities that selective protection of distant neurovascular units may confer meaningful therapeutic benefit in retinal injury due to heart failure even in the presence of incomplete correction of major cardiac lesion.

Additional research should examine the potential function of TGF-β2 in other conditions. Particular attention should be paid to any potential effects in central nervous system disease, including Alzheimer’s disease and multiple sclerosis where TGF-β2 actions are not well understood 43, 44. Moreover, it would be of interest to assess the interaction of TGFβ2 with other signaling pathways, such as Wnt/β-catenin and PI3K/Akt, to better comprehend the regulating networks associated with systemic organ damage 45-48. Therapeutic strategies targeting TGFβ2 and its downstream pathways may help decrease damage from heart failure to the retina and other distant organ systems. The development of these studies will not only bolster the theoretical framework for novel treatment strategies but also offer new avenues for addressing heart failure issues.

In conclusion, our findings provide an alternative strategy for targeting TGFβ2 in retinal damage caused by HF. When TGFβ2 is targeted early, it can block EC senescence, microglial activation and retinal degeneration. This therapeutic approach can offer better outcomes for patients and decrease systemic damage caused by heart failure. Thus, it presents an important opportunity for clinical use.

## Supplementary Material

Supplementary figures.

## Data Availability

The single-nucleus RNA sequencing data used in this study are available from the European Genome-Phenome Archive (EGAS00001006374). The RNA-seq data are available from the Gene Expression Omnibus (GSE116250). All other data supporting the findings of this study are included in the manuscript and supplementary materials or are available from the corresponding author upon reasonable request.

## Abbreviations

cKO: conditional knockout
CUT&Tag: cleavage under targets and tagmentation
EP300: E1A binding protein p300
ERG: electroretinography
EV: extracellular vesicle
GCL: ganglion cell layer
GO: Gene Ontology
GSEA: Gene Set Enrichment Analysis
H3K9ac: histone H3 lysine 9 acetylation
HF: heart failure
HRMEC: human retinal microvascular endothelial cell
HUVEC: human umbilical vein endothelial cell
IFN-γ: interferon gamma
IgG: immunoglobulin G
IL-1β: interleukin-1 beta
IL-6: interleukin-6
INL: inner nuclear layer
IPL: inner plexiform layer
LVEF: left ventricular ejection fraction
LVFS: left ventricular fractional shortening
NVU: neurovascular unit
OCT: optical coherence tomography
ONL: outer nuclear layer
OPL: outer plexiform layer
PBS: phosphate-buffered saline
PCA: principal component analysis
pSMAD2: phosphorylated SMAD2
SA-β-gal: senescence-associated β-galactosidase
SASP: senescence-associated secretory phenotype
snRNA-seq: single-nucleus RNA sequencing
TAC: transverse aortic constriction
TGFβ2: transforming growth factor beta 2
TGFBR2: transforming growth factor beta receptor 2
TNF-α: tumor necrosis factor alpha
UMAP: Uniform Manifold Approximation and Projection
