# Supplementary material for: Endothelial epigenetic senescence driven microglial activation mediates cardio-retinal neuroinflammation in heart failure
Source: Theranostics. 2026 Jul 13;16(14):8055–76. doi: 10.7150/thno.136220 (PMC13440472; doi:10.7150/thno.136220)
Supplement: Supplementary file 1 [file thnov16p8055s1.pdf]

Supplemental Material

Endothelial epigenetic senescence driven microglial activation mediates cardio-retinal neuroinflammation in heart failure

Mengdan Wang, Shuo Zhang, Tursunjan Aziz, Shiyao Zhang, Xiao Wu, Gang Li, Yan Wang

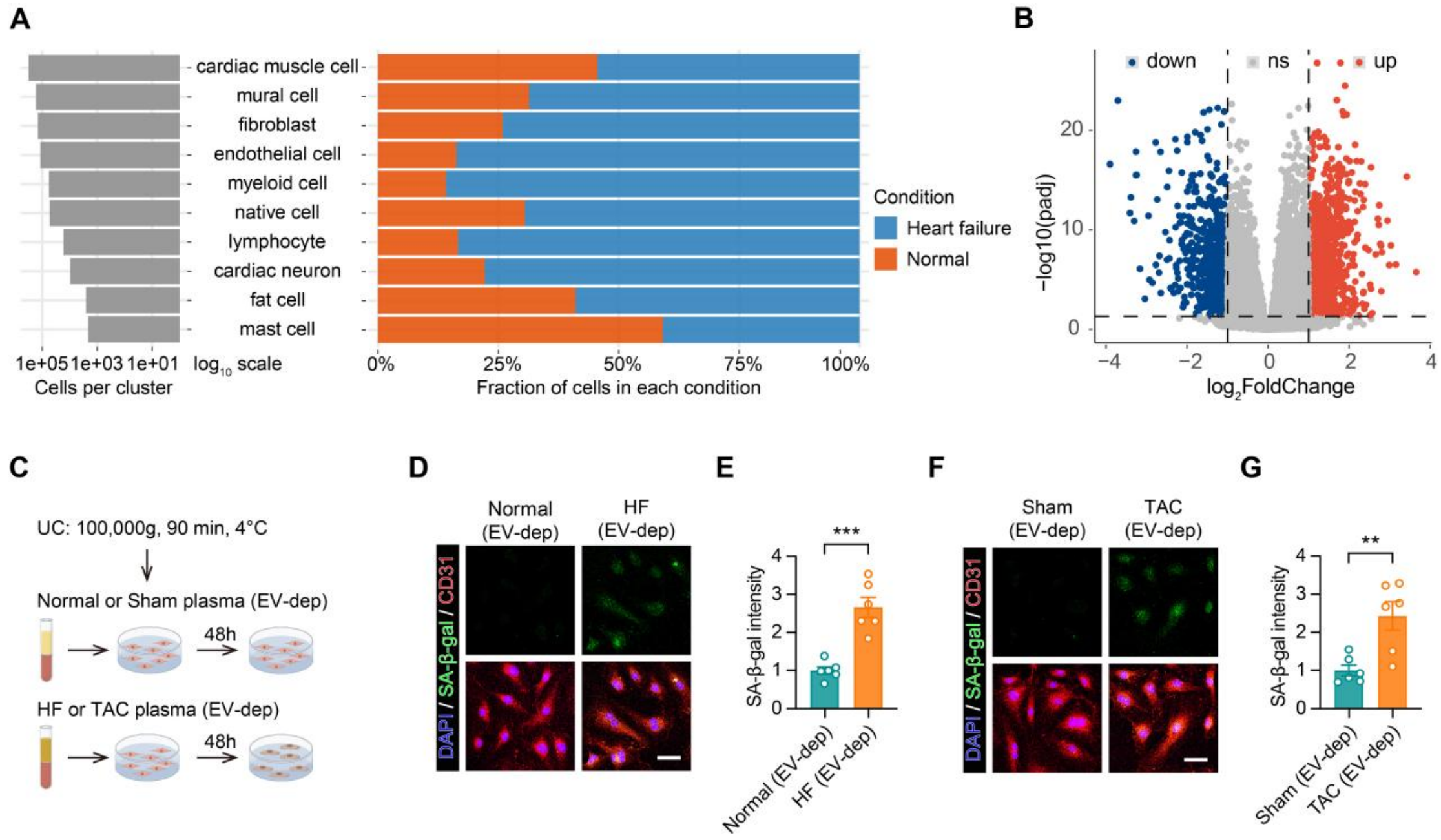

Figure S1. Distribution of cardiac cell populations, differential gene expression in heart failure, and effects of EV-depleted plasma on endothelial cell senescence

(A) Left panel shows the number of cells in each cardiac cell population. Right panel shows the percentage distribution of each cell population under heart failure (blue) and normal (orange) conditions.

(B) Volcano plot showing differential gene expression between heart failure and normal groups. Blue dots represent downregulated genes, red dots represent upregulated genes, and gray dots indicate genes with no significant change.

(C) Schematic of the experimental workflow for extracellular vesicle (EV) depletion. Plasma from normal/HF patients or Sham/TAC mice was subjected to ultracentrifugation (100,000 g, 90 min, 4°C) to deplete EVs. The EV-depleted supernatant was then used to treat endothelial cells for 48 h.

(D-E) Representative images and quantification of SA-beta-gal staining in HUVECs treated with EV-depleted plasma from normal individuals or HF patients for 48 h. Scale bar, 50 µm. n = 6 independent experiments per group. Scale bar, 50 µm.

(F-G) Representative images and quantification of SA-beta-gal staining in HUVECs treated with EV-depleted plasma from Sham or TAC mice for 48 h. Scale bar, 50 µm. n = 6 independent experiments per group. Scale bar, 50 µm.

All data are presented as mean ± SEM. *P*-values were determined by Student's t-test in (E) and (G). $^{**}p < 0.01$, $^{***}p < 0.001$.

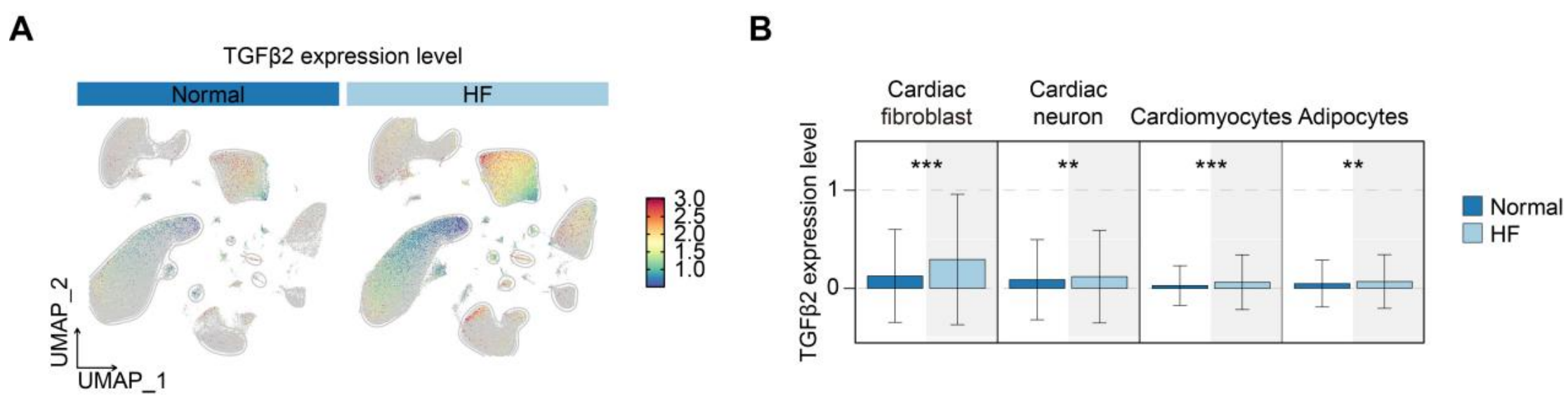

Figure S2. Increased TGFβ2 expression across multiple cardiac cell populations in heart failure

(A) UMAP plots showing the spatial distribution of TGFβ2 expression in cardiac tissues from normal controls and heart failure patients. Color intensity represents the relative TGFβ2 expression level.

(B) Quantitative comparison of TGFβ2 expression in different cardiac cell types between normal controls and heart failure patients, including cardiac fibroblasts, cardiac neurons, cardiomyocytes, and adipocytes. TGFβ2 expression was significantly increased in these cell populations in heart failure samples.

All data are presented as mean ± SD. *P*-values were determined by Student's t-test in (B). $^{**}p < 0.01$, $^{***}p < 0.001$.

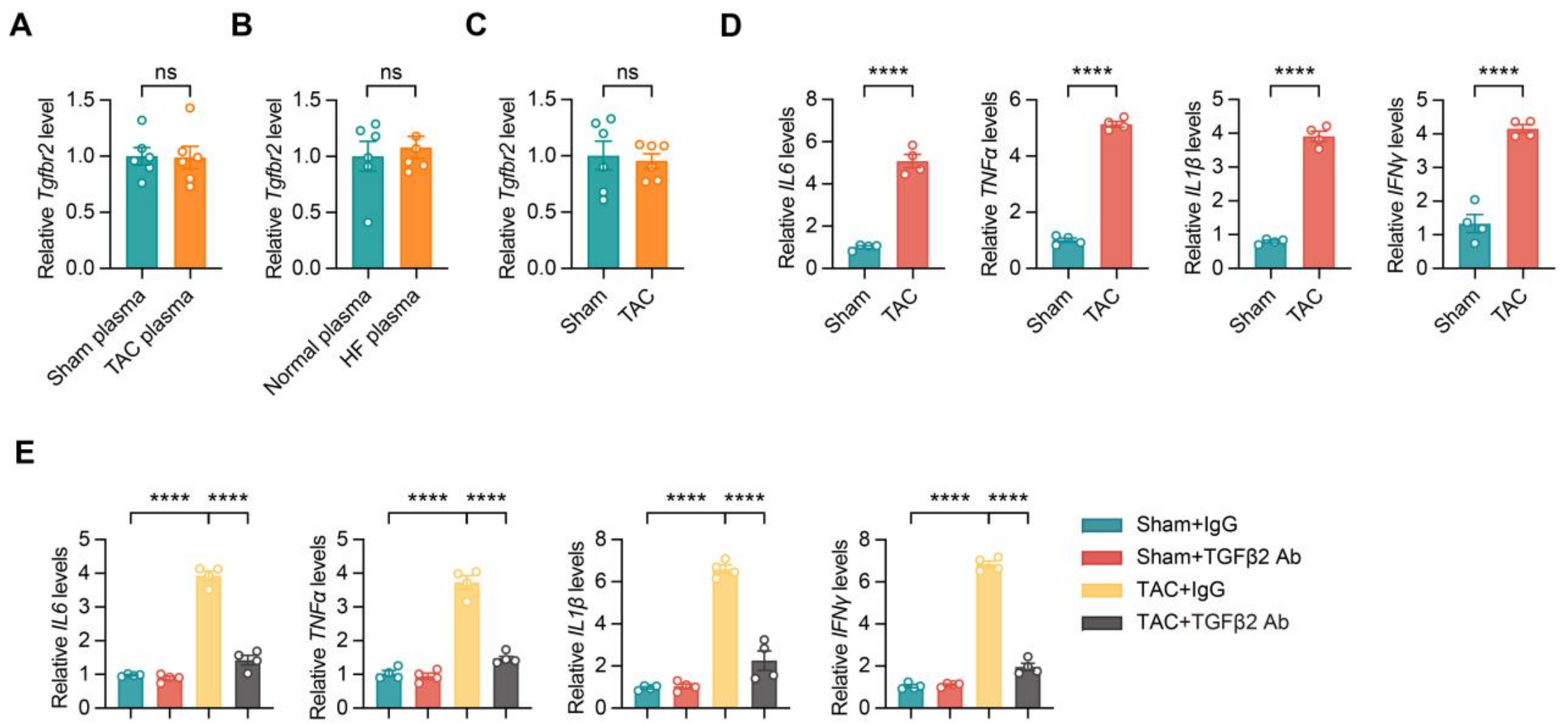

Figure S3. TGFBR2 expression in endothelial cells is unaltered under heart failure conditions, and anti-TGFβ2 antibody ameliorates microglial hyperactivation by inhibiting endothelial cell senescence

(A) Quantitative PCR analysis showing relative *Tgfbr2* mRNA levels in HUVECs treated with plasma from Sham or TAC mice for 48 h, n = 6 independent experiments per group.

(B) Quantitative PCR analysis showing relative *Tgfbr2* mRNA levels in HUVECs treated with plasma from healthy controls or heart failure patients for 48 h, n = 6 independent experiments per group.

(C) Quantitative PCR analysis showing relative *Tgfbr2* mRNA levels in flow cytometry-sorted retinal vascular endothelial cells ($CD31^+CD45^-$) from Sham and TAC mice, n = 6 mice per group.

(D) Quantitative PCR analysis showing the relative expression levels of *IL-6*, *TNF*-α, *IL-1β*, and *IFN-γ* in microglia treated with conditioned medium from endothelial cells exposed to Sham or TAC plasma, n = 4 independent experiments per group.

(E) Quantitative PCR analysis showing the relative expression levels of *IL-6*, *TNF-α*, *IL-1β*, and *IFN-γ* in microglia treated with conditioned medium from endothelial cells exposed to Sham or TAC plasma with IgG or anti-TGFβ2 antibody co-treatment, n = 4 independent experiments per group.

All data are presented as mean ± SEM. *P*-values were determined by Student's t-test in (A-D), and by one-way ANOVA with Dunnett's post hoc analysis in (E). ns, not significant. ****$p < 0.0001$.

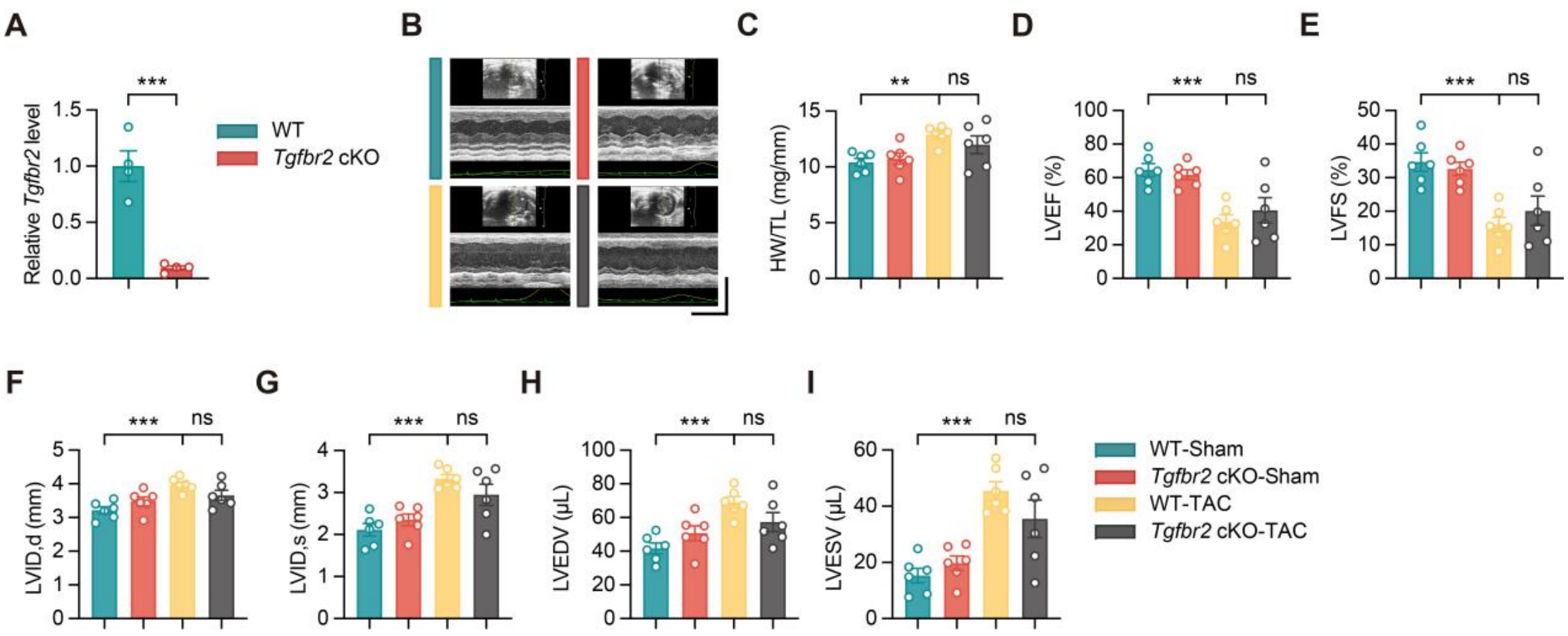

Figure S4. Validation of endothelial-specific *Tgfbr2* deletion and cardiac phenotypes in TAC mice

(A) Quantitative PCR analysis of relative *Tgfbr2* expression in flow cytometry-sorted retinal microvascular endothelial cells isolated from WT and *Tgfbr2* cKO mice after

tamoxifen induction, validating the efficiency of endothelial-specific *Tgfbr2* deletion, n = 4 mice per group.

(B) Representative echocardiographic images from WT-Sham, *Tgfbr2* cKO-Sham, WT-TAC, and *Tgfbr2* cKO-TAC mice. Horizontal scale bar, 300 ms; vertical scale bar, 6 mm.

(C-I) Quantification of heart weight/tibia length (HW/TL) ratio (C), left ventricular ejection fraction (LVEF) (D), left ventricular fractional shortening (LVFS) (E), left ventricular internal diameter at end-diastole (LVID,d) (F), left ventricular internal diameter at end-systole (LVID,s) (G), left ventricular end-diastolic volume (LVEDV) (H), and left ventricular end-systolic volume (LVESV) (I) in the indicated groups, n = 6 mice per group.

All data are presented as mean ± SEM. Each dot represents one mouse. *P*-values were determined by Student's t-test in (A) and one-way ANOVA with Dunnett's post hoc analysis in (C-I). ns, not significant. $^{**}p < 0.01$, $^{***}p < 0.001$.

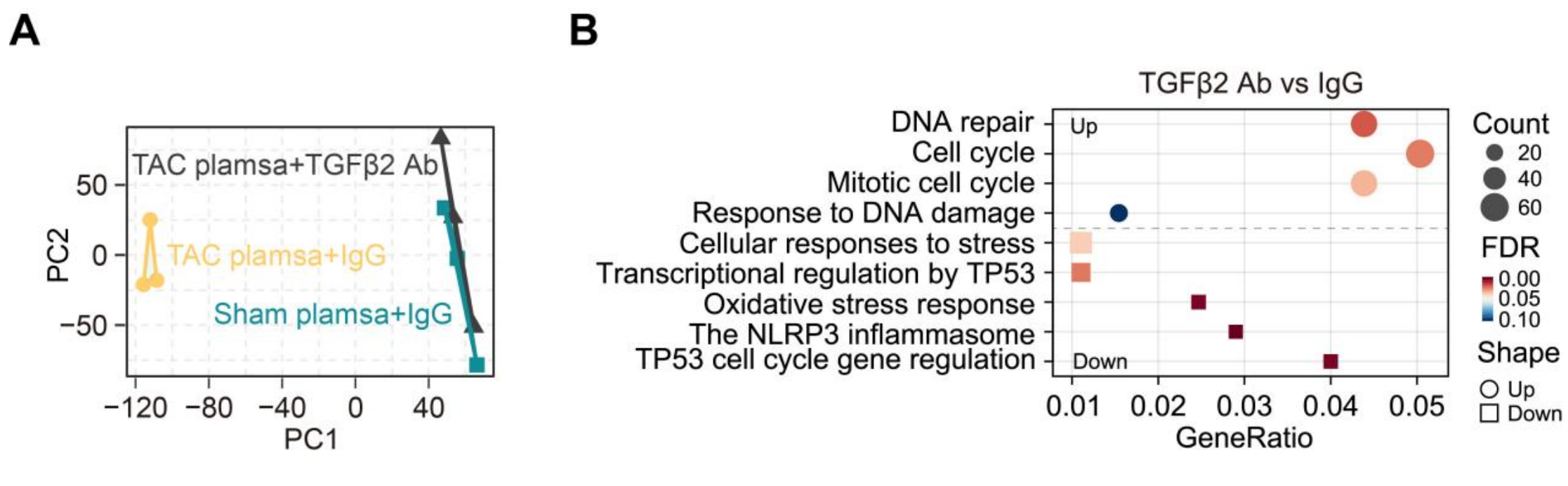

Figure S5. Effects of anti-TGFβ2 antibody on gene expression in endothelial cells treated with TAC plasma

(A) Principal component analysis shows that anti-TGFβ2 antibody significantly improved TAC plasma-induced changes in endothelial cell gene expression, n = 3 independent experiments per group.

(B) Gene Ontology enrichment analysis. Compared to the TAC plasma + IgG group, the anti-TGFβ2 antibody upregulated pathways above the dashed line and downregulated pathways below the dashed line, n = 3 independent experiments per group.
